# Supplementary material for: Allosteric control of olefin isomerization kinetics via remote metal binding and its mechanochemical analysis
Source: Nat Commun. 2023 Aug 21;14:5074. doi: 10.1038/s41467-023-40842-5 (PMC10442431; doi:10.1038/s41467-023-40842-5)
Supplement: Supplementary file 1 — Supplementary Information [file 41467_2023_40842_MOESM1_ESM.pdf]

Allosteric control of olefin isomerization kinetics via remote metal binding and its mechanochemical analysis

Supplementary Information

Yichen Yu,[1$] Robert T O'Neill,[2$] Roman Boulatov,[2*] Ross A. Widenhoefer,[1*] and Stephen L. Craig[1*]

[1]Department of Chemistry, Duke University, Durham, North Carolina 27708, USA.
[2]Department of Chemistry, University of Liverpool, Crown Street, Liverpool L69 7ZD, UK.
$ equal contributions

Contents

Tables of kinetic data	S2
Plots of kinetic data	S3
DFT calculations	S16
Synthesis of bisphosphine complexes	S27
Supplementary References	S28

Supplementary Table 1. First order rate constants for the isomerization of [E(m,n)]$PtCl_2$ (8.6 mM) in DMF-d_7 and for the isomerization of E(m,n) (8.6 mM) in *p*-xylene-d_{10}.

entry	macrocycle	Temp (°C)	$(10^5)k$ (s^{-1})	Suppl. Figure
1	[(*S,R*)-E(2,3)]$PtCl_2$	80	24 ± 2	1
2	[(*S,R*)-E(2,3)]$PtCl_2$	75	12.6 ± 0.6	2
3	[(*S,R*)-E(2,3)]$PtCl_2$	71	7.9 ± 0.9	3
4	[(*S,R*)-E(2,3)]$PtCl_2$	67	5.6 ± 0.2	4
5	[(*S,R*)-E(2,3)]$PtCl_2$	64	3.5 ± 0.2	5
6	[(*S,S*)-E(3,3)]$PtCl_2$	125	29 ± 2	7
7	[(*S,R*)-E(3,3)]$PtCl_2$	121	33 ± 2	8
8	[(*S,R*)-E(2,2)]$PtCl_2$	6	57 ± 5	9
9	[(*S,R*)-E(2,2)]$PtCl_2$	3	38.1 ± 1.7	10
10	[(*S,R*)-E(2,2)]$PtCl_2$	–1	24.3 ± 1.4	11
11	[(*S,R*)-E(2,2)]$PtCl_2$	–5	10.9 ± 0.3	12
12	[(*S,R*)-E(2,2)]$PtCl_2$	–10	5.64 ± 0.14	13
13	(*S,R*)-E(2,2)	84	30.5 ± 3.6	14
14	(*S,R*)-E(2,2)	78	14.3 ± 0.8	15
15	(*S,R*)-E(2,2)	74	10.2 ± 0.6	16
16	(*S,R*)-E(2,2)	69	4.91 ± 0.14	17
17	(*S,R*)-E(2,3)	140	76 ± 5	18
18	(*S,R*)-E(2,3)	135	46 ± 4	19
19	(*S,R*)-E(2,3)	130	31.1 ± 1.6	20
20	(*S,R*)-E(2,3)	127	22 ± 2	21
21	(*S,R*)-E(2,3)	123	13.4 ± 0.8	22
22	(*S,R*)-E(2,3)	120	9.3 ± 0.3	25
23	(*S,R*)-E(3,3)	128	9.1 ± 0.5	23
24	(*S,S*)-E(3,3)	131	8.9 ± 0.4	24

Supplementary Table 2. Activation parameters for isomerization of [E(m,n)]$PtCl_2$ in DMF-d_7 and for the isomerization of E(m,n) in *p*-xylene-d_{10}.

macrocycle	T (°C)	$\Delta H^‡$ (kcal/mol)	$\Delta S^‡$ (e.u.)	$\Delta G^‡_{298\ K}$ (kcal/mol)	Suppl. Figure
[(*S,R*)-E(2,3)]$PtCl_2$	64 - 80	26.8 ± 1.1	0.5 ± 2.5	26.7 ± 1.9	6
[(*S,R*)-E(2,2)]$PtCl_2$	–10 - 6	21.6 ± 1.0	4.4 ± 3.5	20.3 ± 2.0	6
(*S,R*)-E(2,2)	69 - 84	28.4 ± 1.1	4.8 ± 3.1	27.0 ± 2.0	6
(*S,R*)-E(2,3)	120 - 140	32.8 ± 1.2	6.0 ± 2.9	31.0 ± 2.0	6

Plots of Kinetic Data

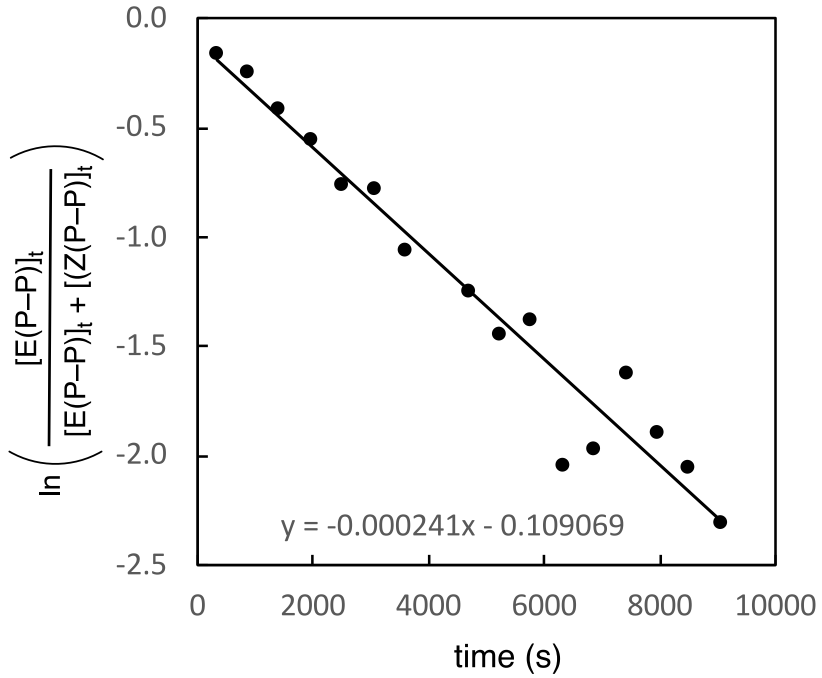

Supplementary Figure 1. First order rate plot for the isomerization of [(*S,R*)-E(2,3)]$PtCl_2$ (8.61 mM) in DMF-d_7 at 80 °C.

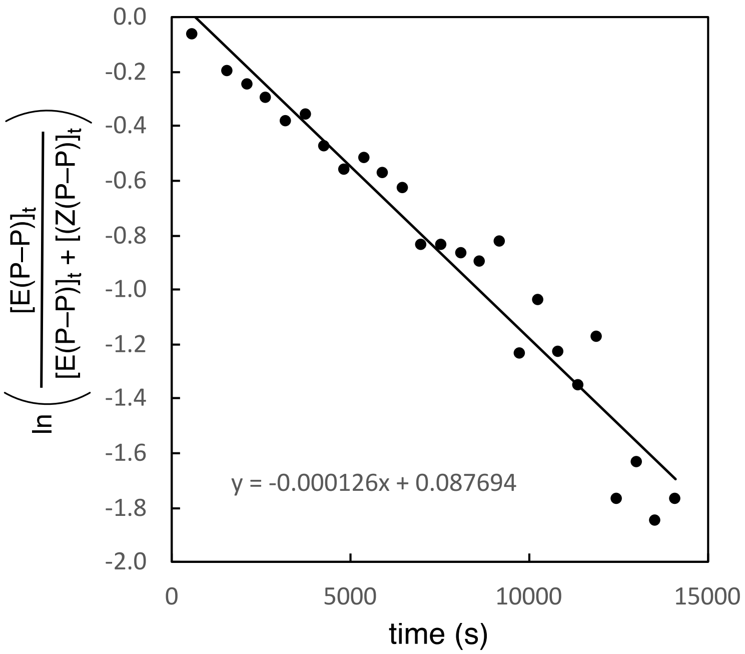

Supplementary Figure 2. First order rate plot for the isomerization of [(*S,R*)-E(2,3)]$PtCl_2$ (8.61 mM) in DMF-d_7 at 75 °C.

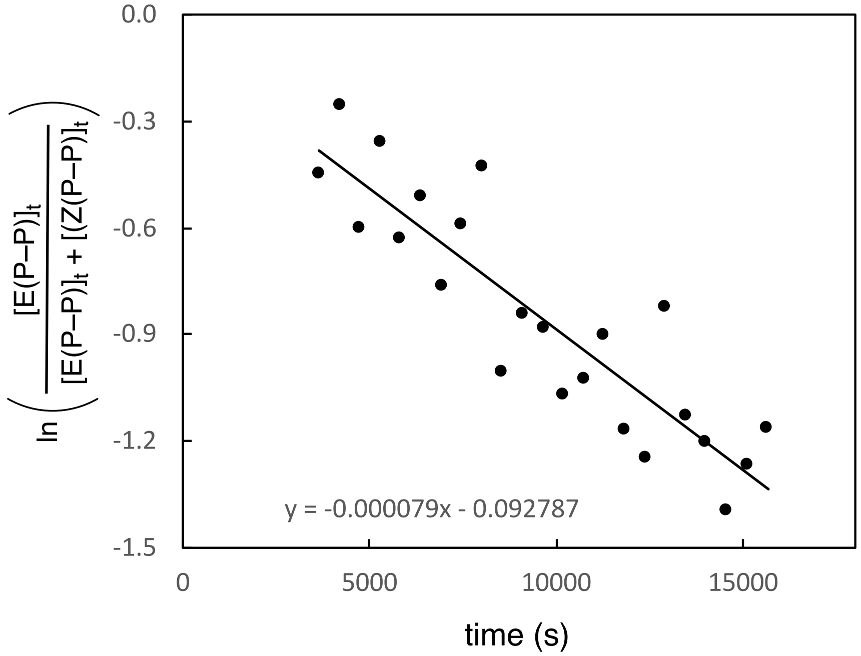

Supplementary Figure 3. First order rate plot for the isomerization of [(*S,R*)-E(2,3)]$PtCl_2$ (8.61 mM) in DMF-d_7 at 71 °C.

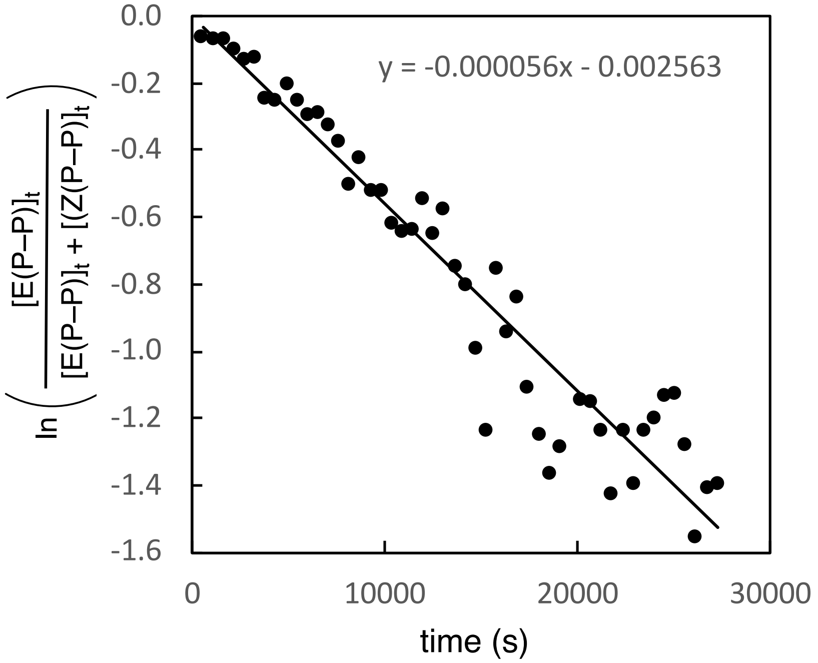

Supplementary Figure 4. First order rate plot for the isomerization of [(*S,R*)-E(2,3)]$PtCl_2$ (8.61 mM) in DMF-d_7 at 67 °C.

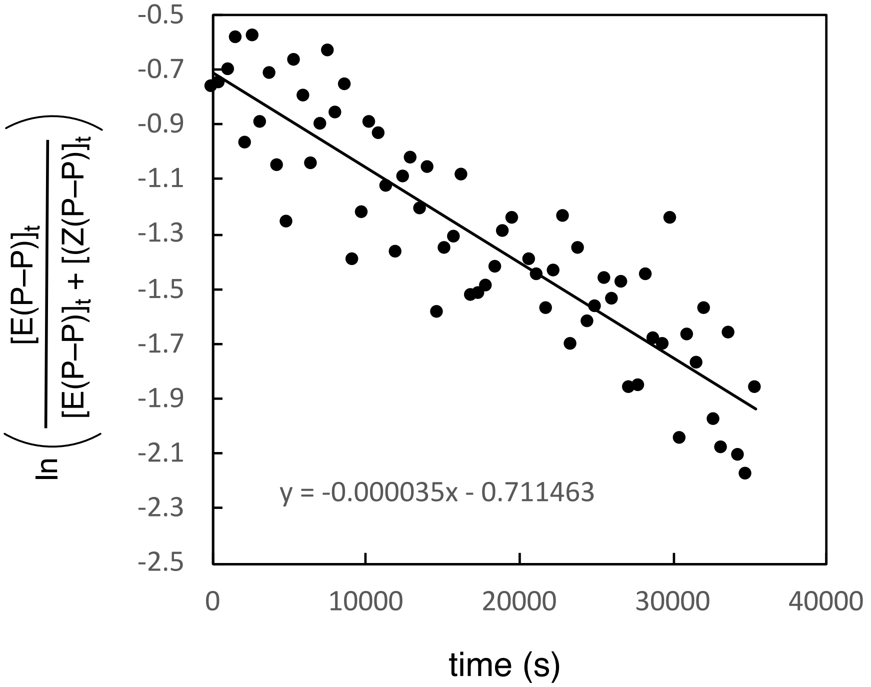

Supplementary Figure 5. First order rate plot for the isomerization of [(*S,R*)-E(2,3)]$PtCl_2$ (8.61 mM) in DMF-d_7 at 64 °C.

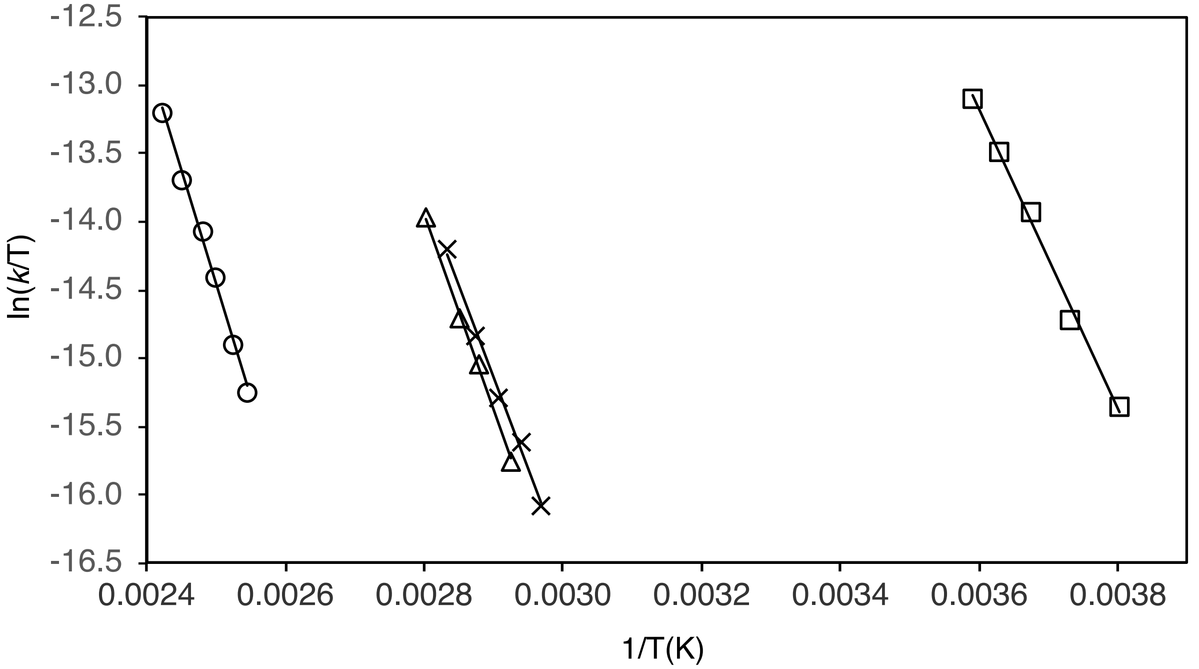

Supplementary Figure 6. Eyring plots for the isomerization of [(*S,R*)-E(2,3)]$PtCl_2$ (×), [(*S,R*)-E(2,2)]$PtCl_2$ (□), (*S,R*)-E(2,3) (○), (*S,R*)-E(2,2) (Δ).

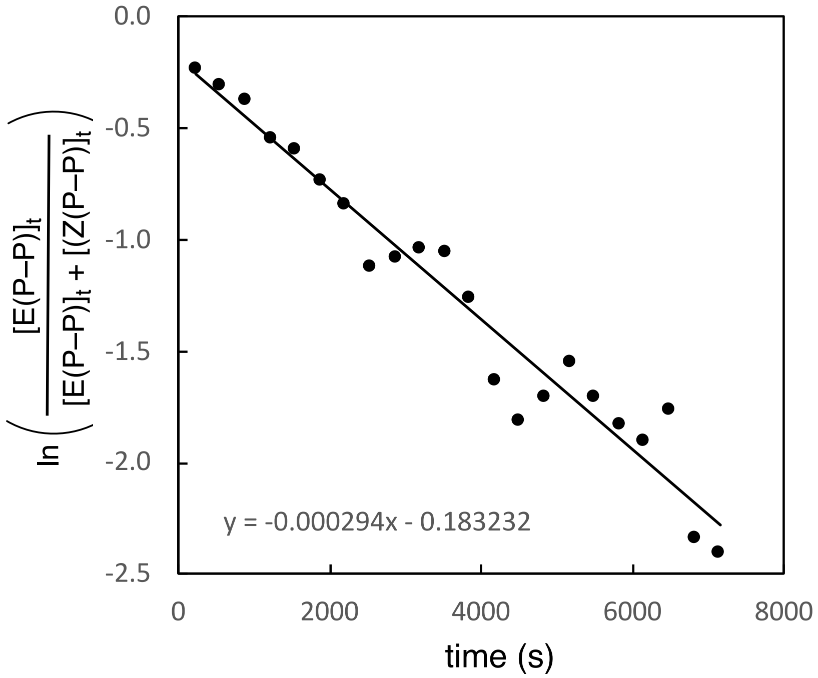

Supplementary Figure 7. First order rate plot for the isomerization of (*S,S*)-[E(3,3)]$PtCl_2$ (8.61 mM) in DMF-d_7 at 125 °C.

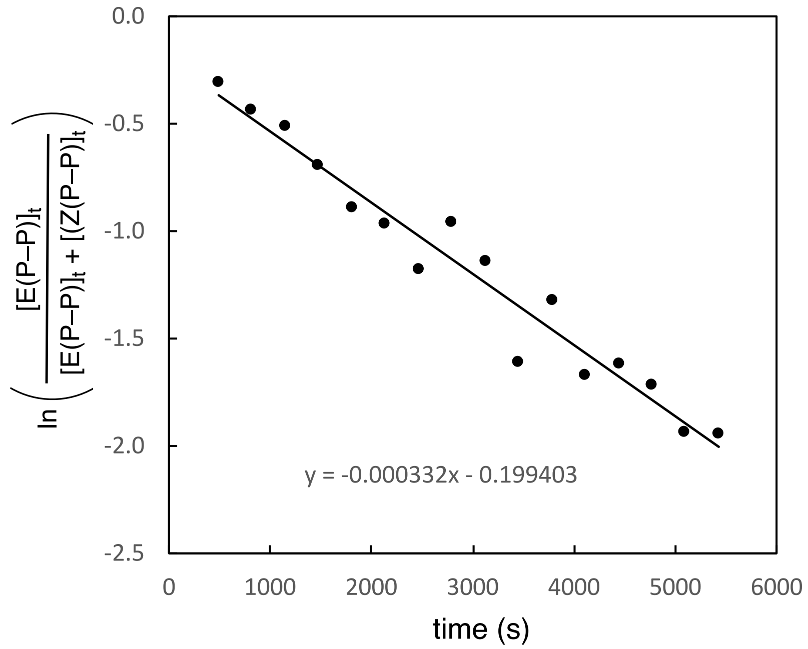

Supplementary Figure 8. First order rate plot for the isomerization of [(*S,R*)-E(3,3)]$PtCl_2$ (8.61 mM) in DMF-d_7 at 121 °C.

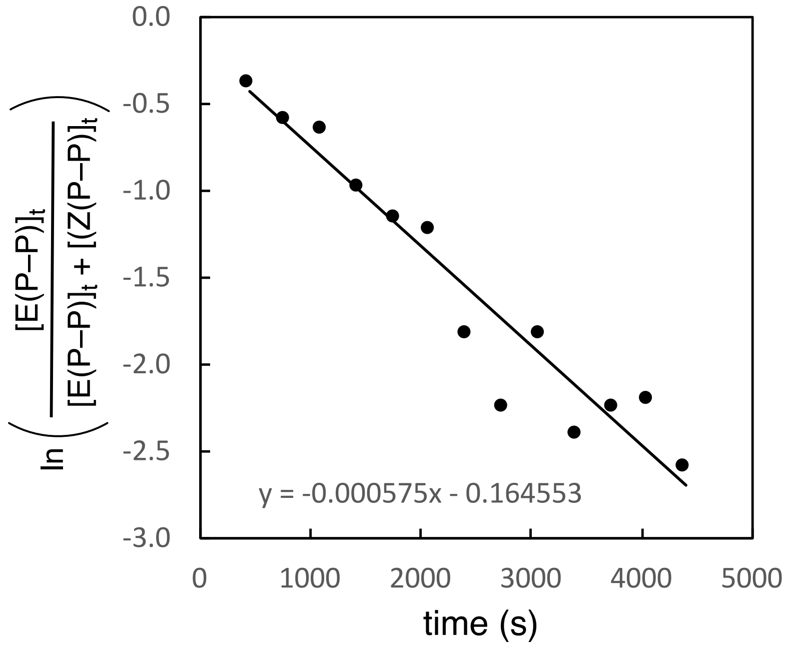

Supplementary Figure 9. First order rate plot for the isomerization of [(*S,R*)-E(2,2)]$PtCl_2$ (8.61 mM) in DMF-d_7 at 6 °C.

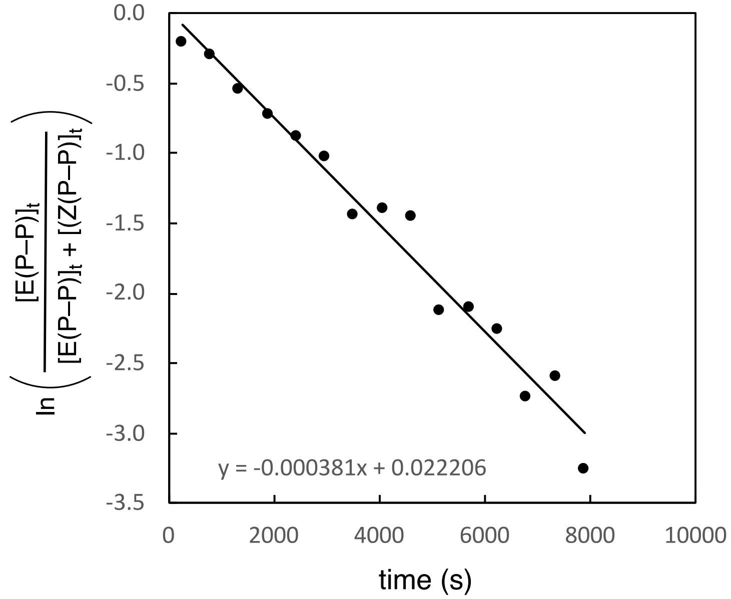

Supplementary Figure 10. First order rate plot for the isomerization of [(*S,R*)-E(2,2)]$PtCl_2$ (8.61 mM) in DMF-d_7 at 3 °C.

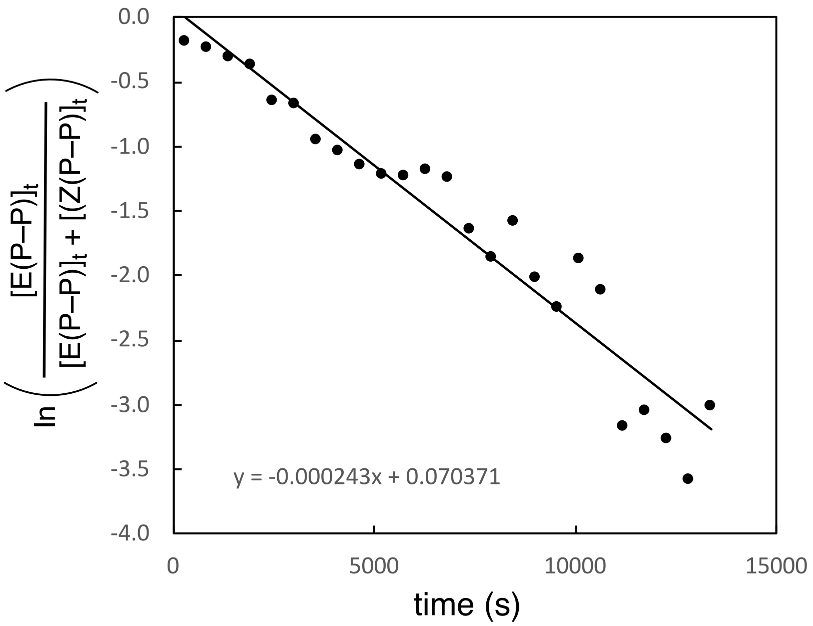

Supplementary Figure 11. First order rate plot for the isomerization of [(*S,R*)-E(2,2)]$PtCl_2$ (8.61 mM) in DMF-d_7 at -1 °C.

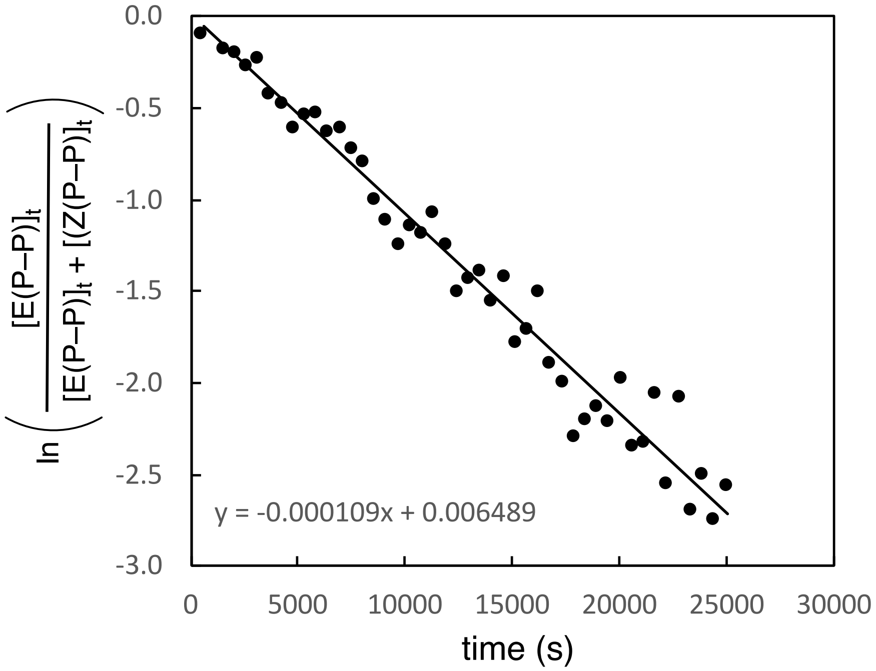

Supplementary Figure 12. First order rate plot for the isomerization of [(*S,R*)-E(2,2)]$PtCl_2$ (8.61 mM) in DMF-d_7 at –5 °C.

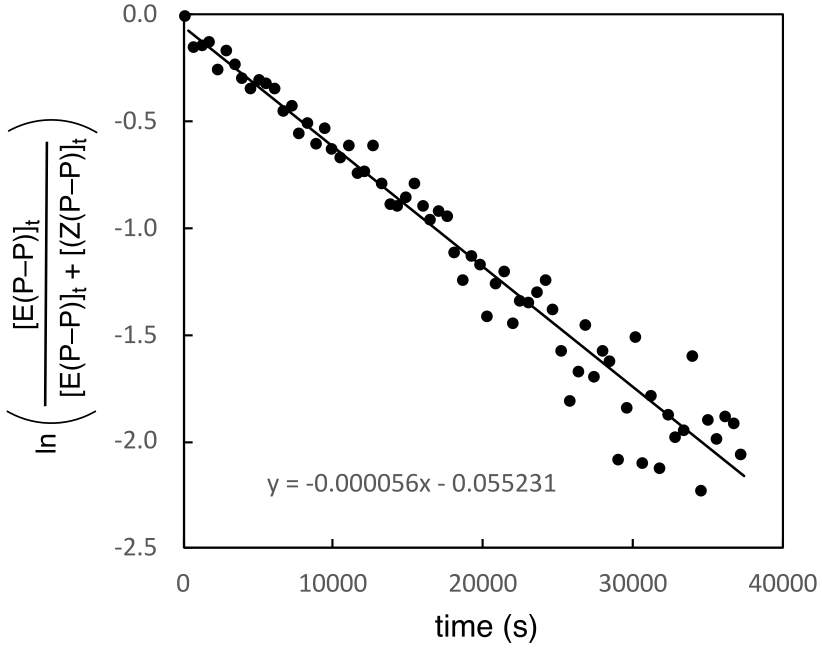

Supplementary Figure 13. First order rate plot for the isomerization of [(*S,R*)-E(2,2)]$PtCl_2$ (8.61 mM) in DMF-d_7 at –10 °C.

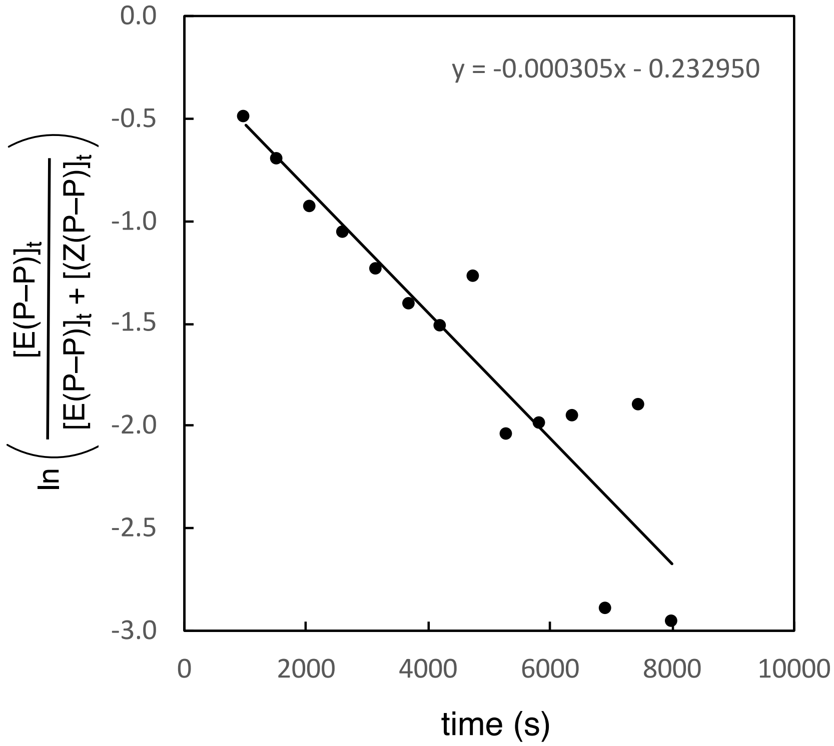

Supplementary Figure 14. First order rate plot for the isomerization of (*S,R*)-E(2,2) (8.61 mM) in *p*-xylene-d_{10} at 84 °C.

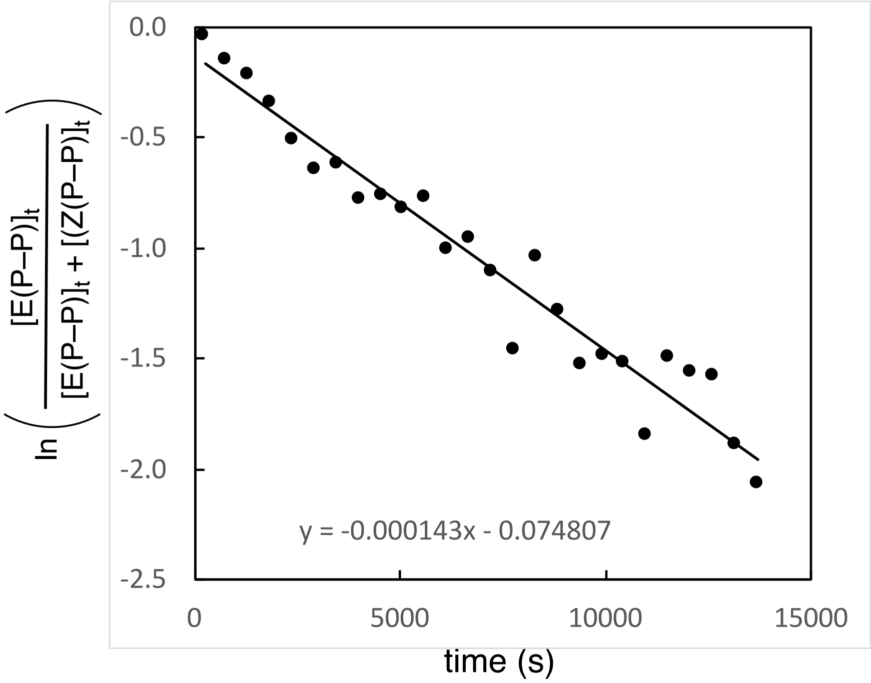

Supplementary Figure 15. First order rate plot for the isomerization of (*S,R*)-E(2,2) (8.61 mM) in *p*-xylene-d_{10} at 78 °C.

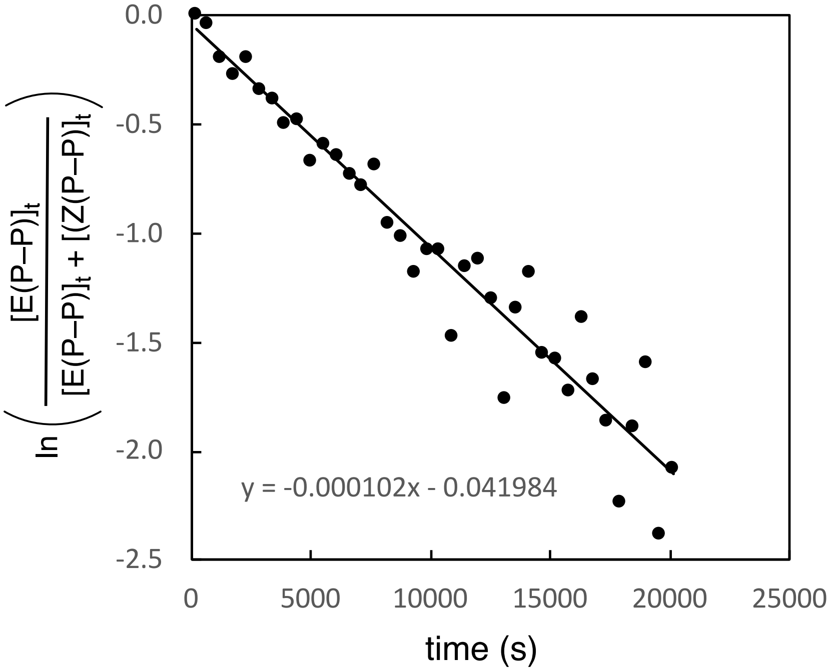

Supplementary Figure 16. First order rate plot for the isomerization of (*S,R*)-E(2,2) (8.61 mM) in *p*-xylene-d_{10} at 74 °C.

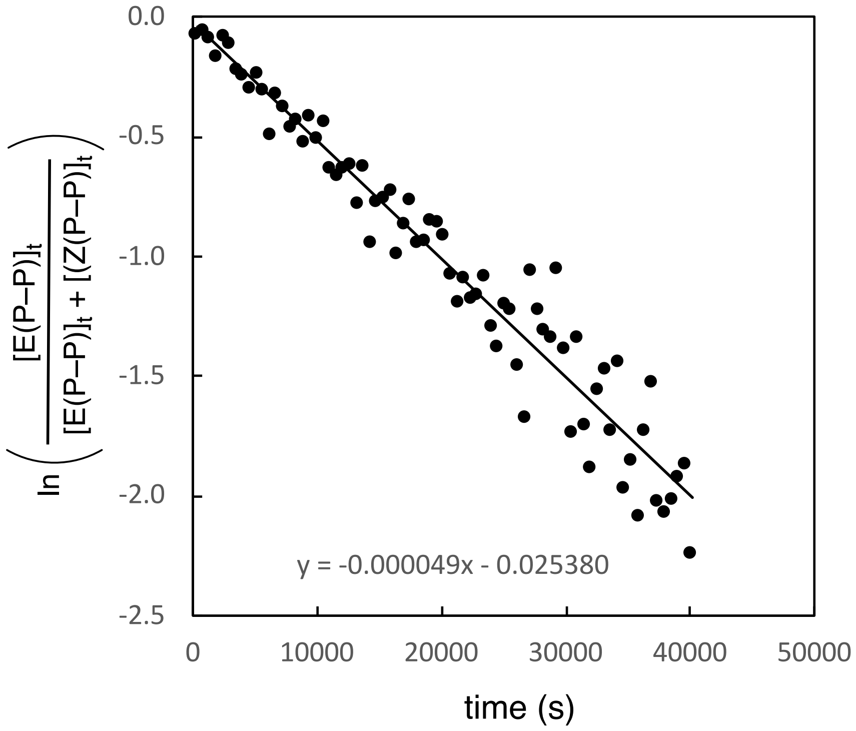

Supplementary Figure 17. First order rate plot for the isomerization of (*S,R*)-E(2,2) (8.61 mM) in *p*-xylene-d_{10} at 69 °C.

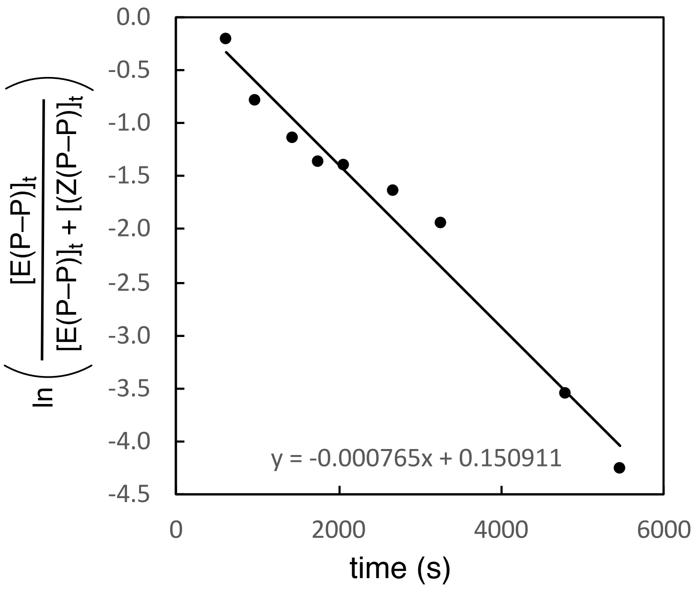

Supplementary Figure 18. First order rate plot for the isomerization of (*S,R*)-E(2,3) (8.61 mM) in *p*-xylene-d_{10} at 140 °C.

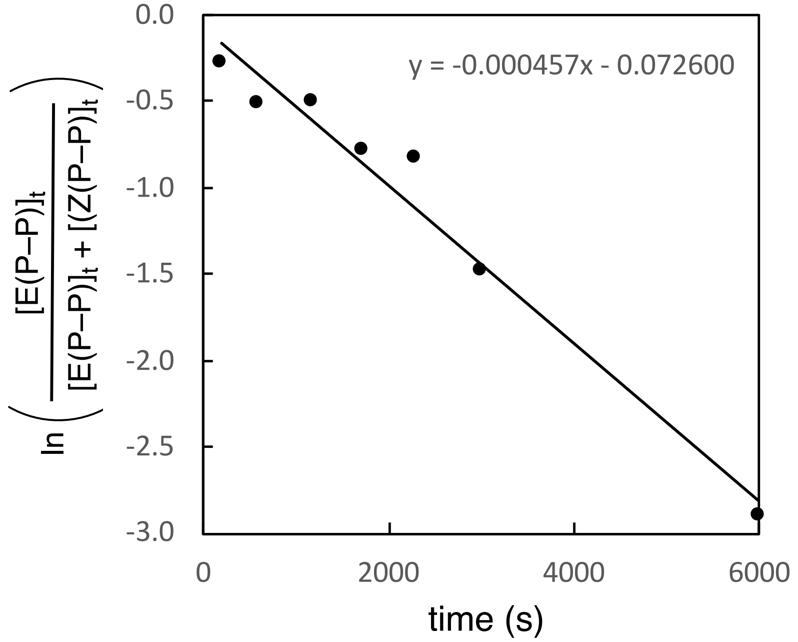

Supplementary Figure 19. First order rate plot for the isomerization of (*S,R*)-E(2,3) (8.61 mM) in *p*-xylene-d_{10} at 135 °C.

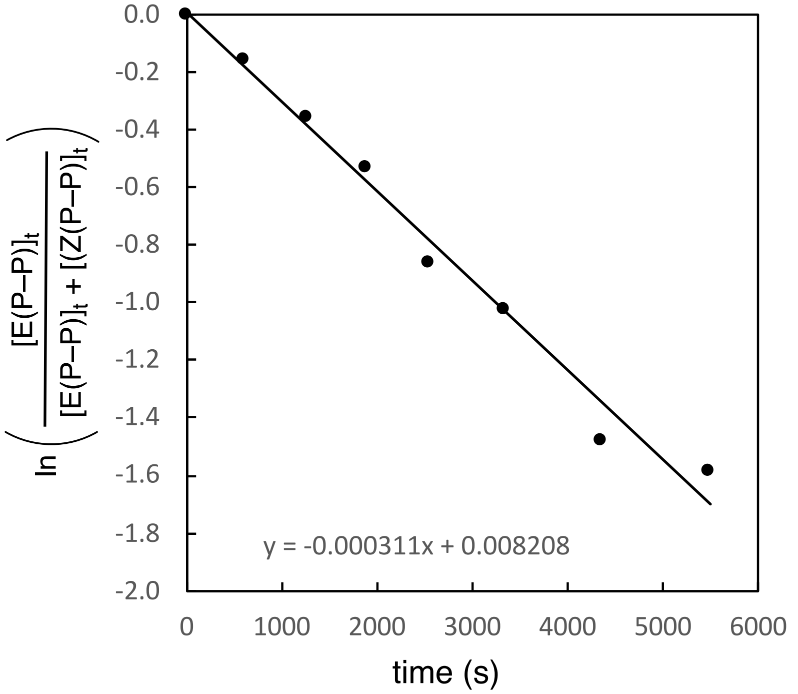

Supplementary Figure 20. First order rate plot for the isomerization of (*S,R*)-E(2,3) (8.61 mM) in *p*-xylene-d_{10} at 130 °C.

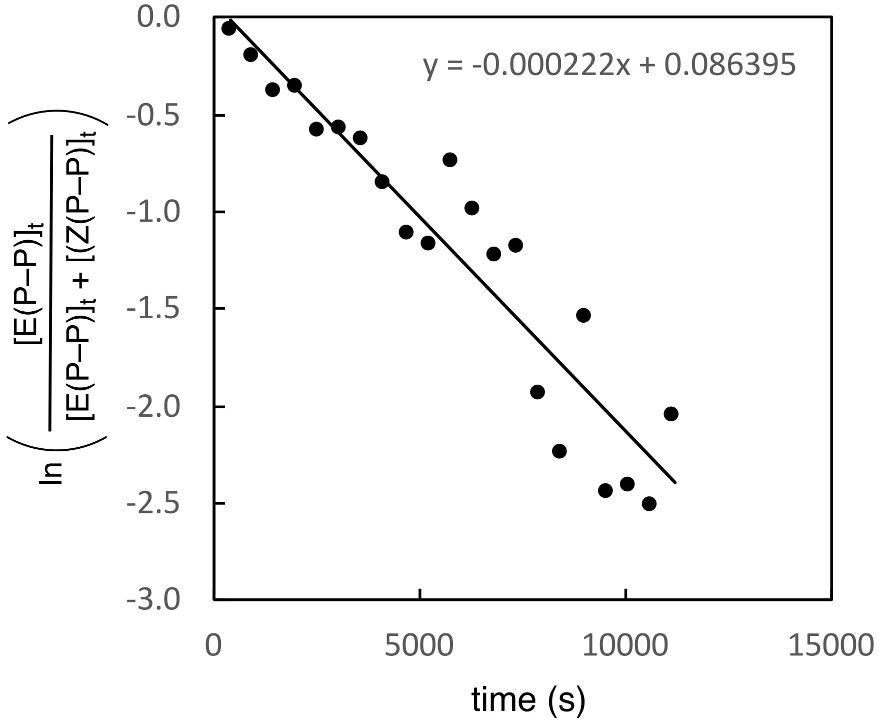

Supplementary Figure 21. First order rate plot for the isomerization of (*S,R*)-E(2,3) (8.61 mM) in *p*-xylene-d_{10} at 127 °C.

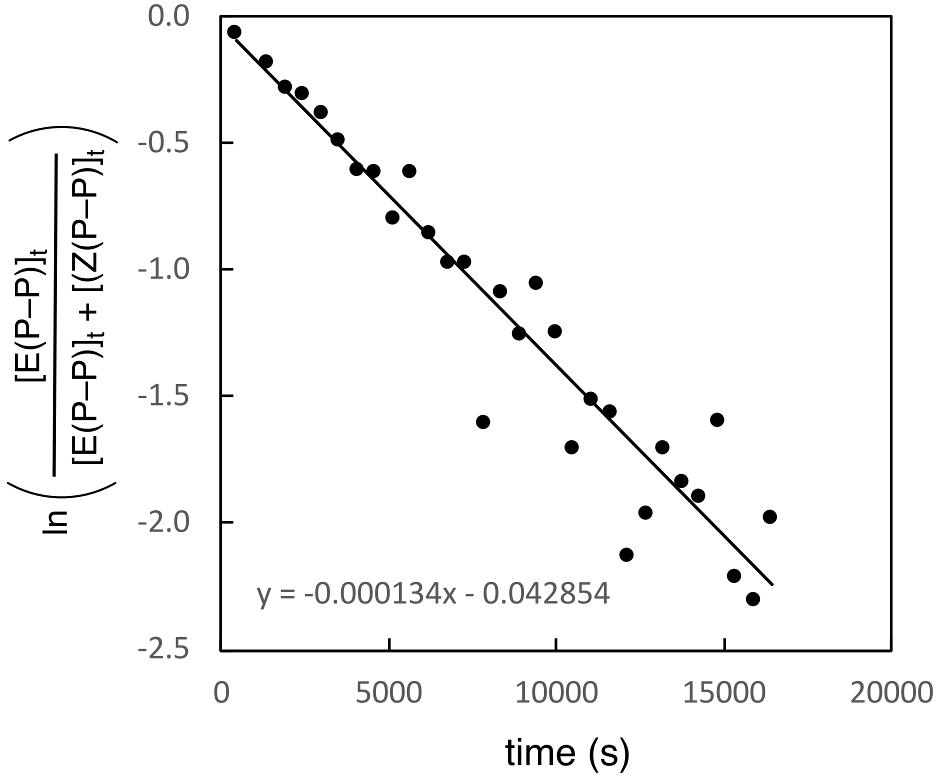

Supplementary Figure 22. First order rate plot for the isomerization of (*S,R*)-E(2,3) (8.61 mM) in *p*-xylene-d_{10} at 123 °C.

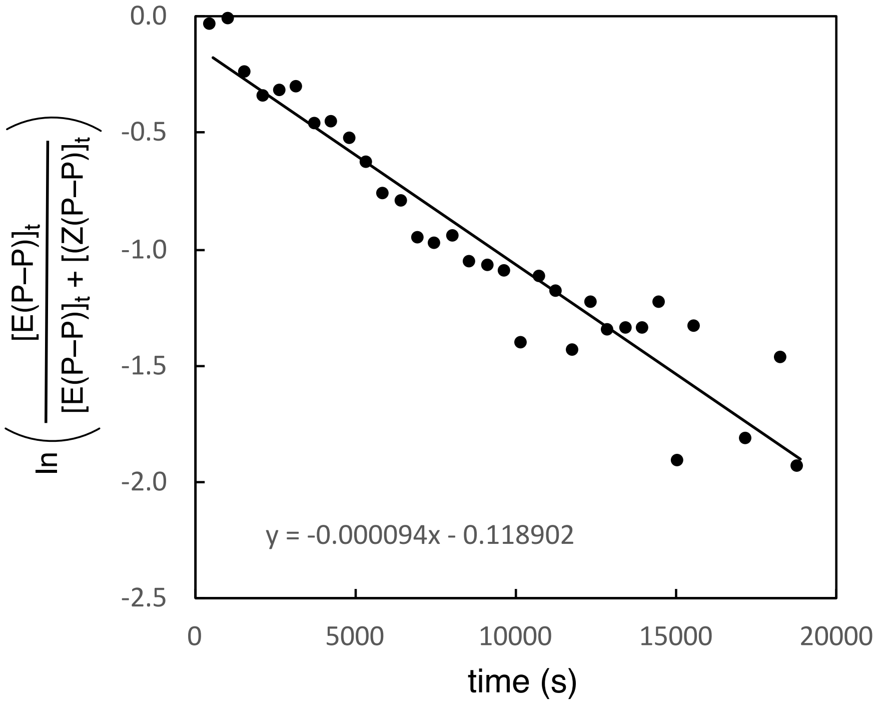

Supplementary Figure 23. First order rate plot for the isomerization of (*S,R*)-E(3,3) (8.61 mM) in *p*-xylene-d_{10} at 128 °C.

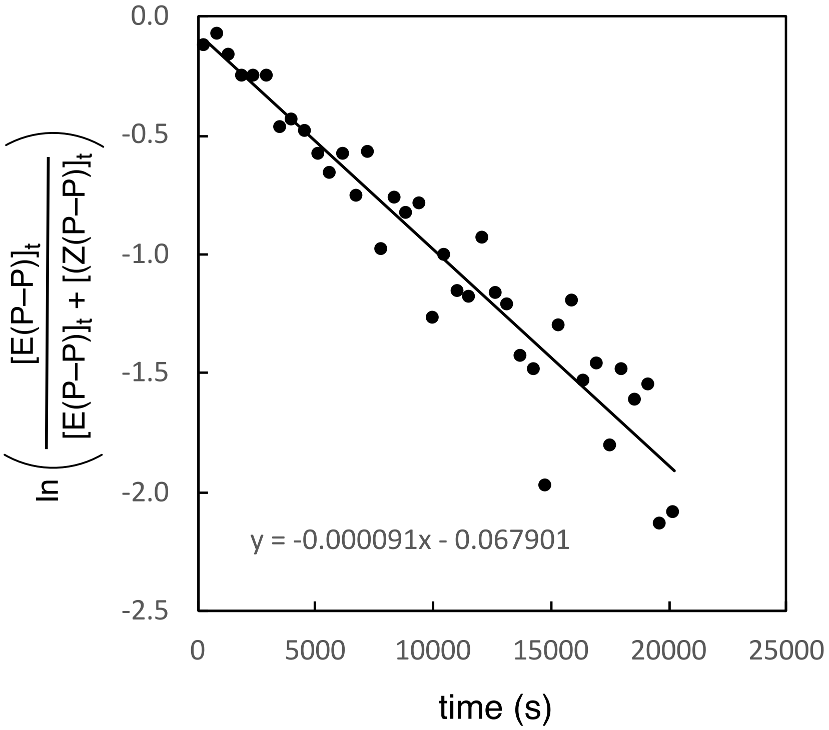

Supplementary Figure 24. First order rate plot for the isomerization of (*S,S*)-E(3,3) (8.61 mM) in *p*-xylene-d_{10} at 131 °C.

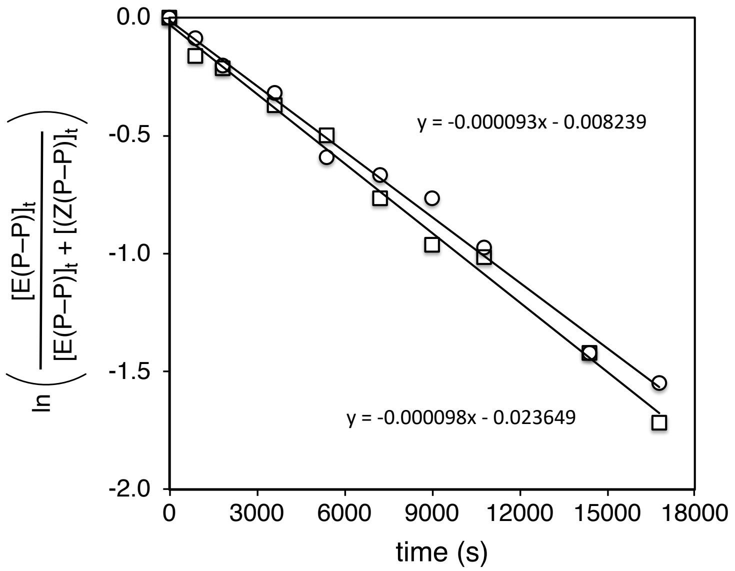

Supplementary Figure 25. First order rate plots for the isomerization of (*S*,*R*)-E(2,3) (8.61 mM) in *p*-xylene-d_{10} (○) and DMF-d_7 (□) at 120 °C.

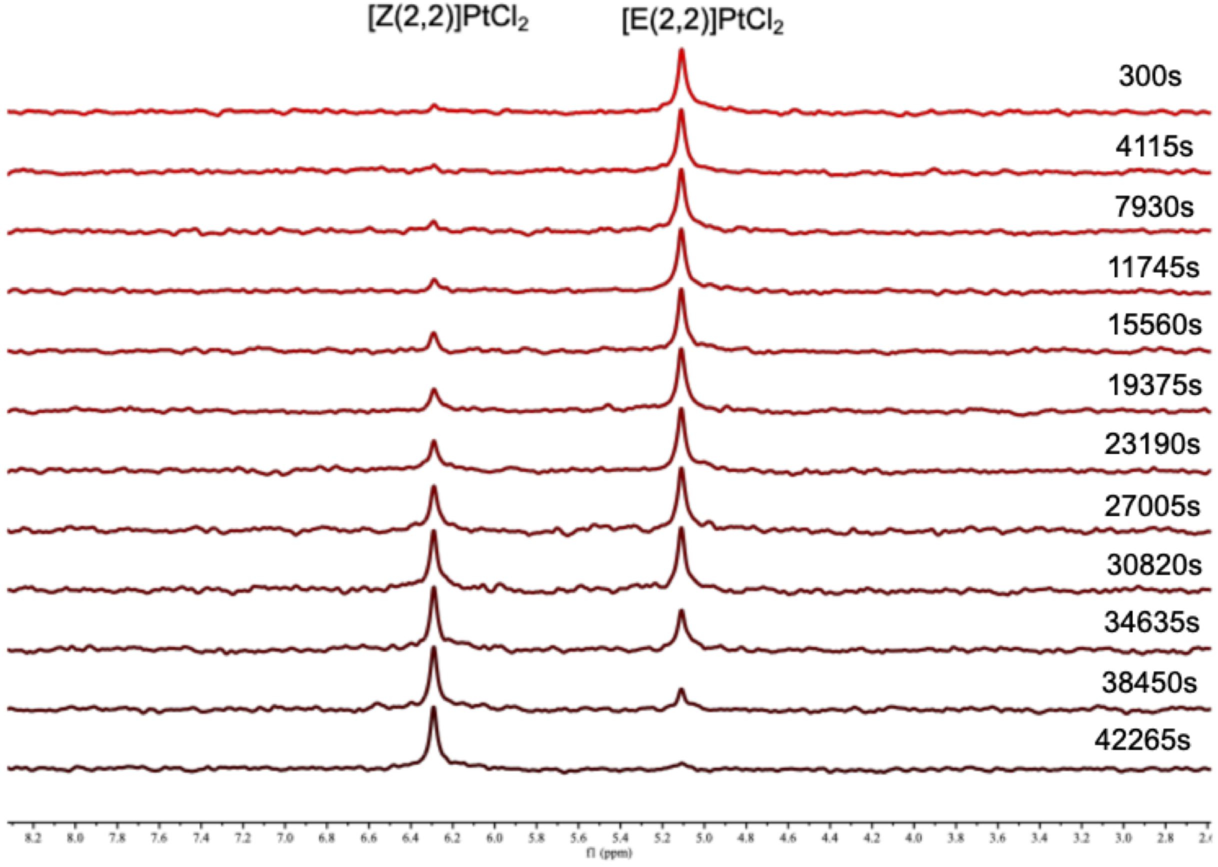

Supplementary Figure 26. ^{31}P NMR spectra for the isomerization of [(*S*,*R*)-E(2,2)]$PtCl_2$ (8.61 mM) in DMF-d_7 at –10 °C.

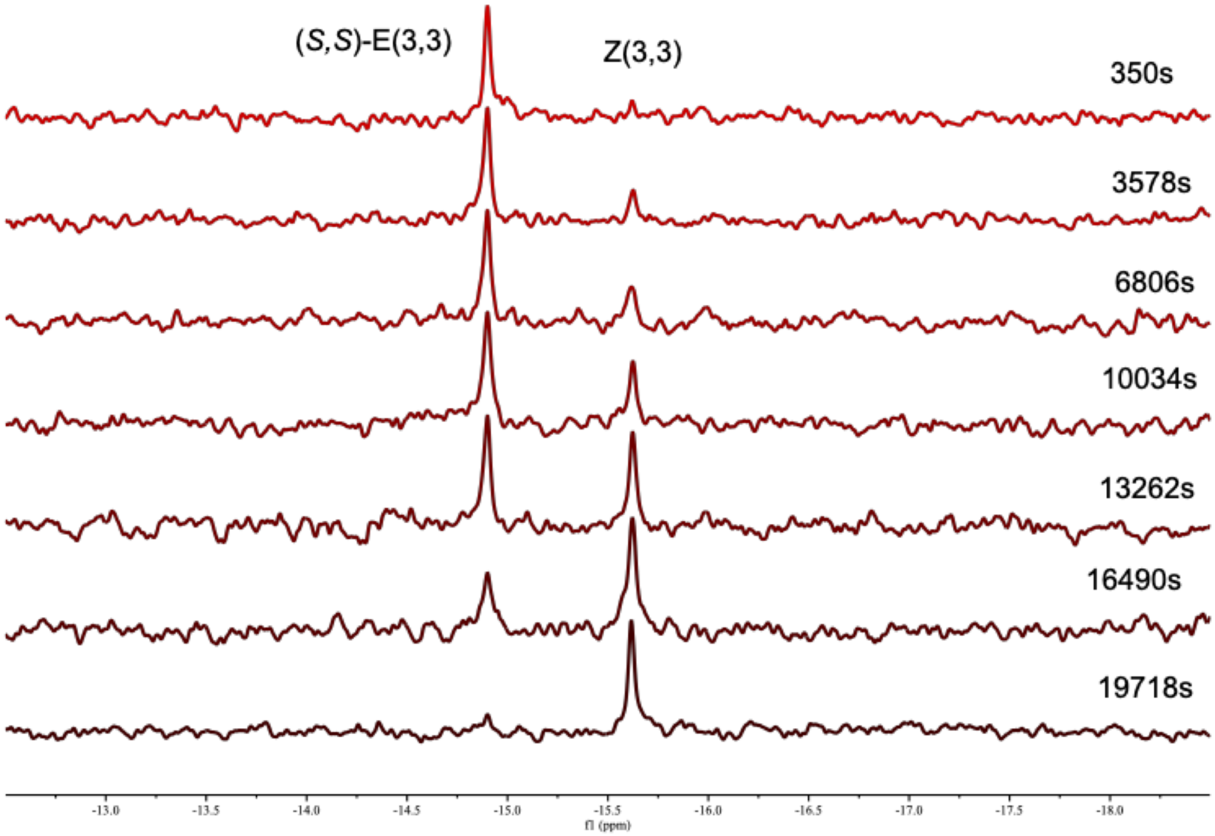

Supplementary Figure 27. ^{31}P NMR spectra for the isomerization of (*S,S*)-E(3,3) (8.61 mM) in *p*-xylene-d_{10} at 131 °C.

DFT calculations and mechanochemical model.

Methods. All calculations were performed with Gaussian 16.C in vacuum; the Berny algorithm was applied to locate stationary points and tight convergence criteria and ultrafine integration grids were used in optimizations and frequency calculations. Reactant conformers of the macrocycles were generated and optimized as previously described,[1] except for the used model chemistry (B3LYP/def2SVP); we previously demonstrated that the def2SVP basis set leads to faster convergence of geometry optimizations of complexes of heavy transition metals and yields kinetic barriers for reactions of such complexes in better agreement with experiment than LANL2DZ.[2]

The initial guesses for transition-state conformers were generated from optimized conformers of Z and E macrocycles by manually changing the C_{Ar}-C=C-C_{Ar} torsion to 90°, and adjusting the torsions and bond angles of the straps to adopt the molecular geometry of the rest of each conformation to the new C6,C6' distance of the SS moiety. For each candidate geometry the stable wavefunction at uB3LYP/6-31+G* level was generated first with the stable=opt option, followed by geometry optimization to an energy minimum with both torsions of the isomerizing C=C bond constrained to prevent pyramidalization of non-aromatic sp^2 C atoms. Converged geometries were subject to another wavefunction stability test and analytical frequencies were

calculated for all geometries that passed; optimized geometries that corresponded to unstable wavefunctions were subjected to another round of constrained optimization using the new stable wavefunctions. All such secondary optimizations converged to geometries with stable UHF wavefunctions. The output of frequencies calculations was used for Berny optimization to transition state.

Analytical frequencies were calculated on converged geometries of all thermally accessible (i.e., within 1.5 kcal/mol of the corresponding conformational minimum) Pt-free macrocycles and of both diastereomers of (2,2) Pt macrocycles and on the minimum energy conformers of all other Pt macrocycles. Extending frequency calculations to all conformers of Pt (2,3) and (3,3) macrocycles was unaffordably expensive. All calculated frequencies had 0 imaginary modes for minima and one imaginary mode for each TS conformer. Free energies of individual conformers were sums of the single-point electronic energy calculated at the (u)BMK/def2SVP level and the corresponding thermodynamic corrections calculated statistically-mechanically with all frequencies <500 cm^{-1} replaced with 500 cm^{-1} to avoid artifactually large contributions of vibrations to $\Delta G^{\ddagger}$.[3] For Pt macrocycles of (2,3) and (3,3), the thermodynamic corrections derived from frequency calculations on the lowest-energy conformers were used for all thermally accessible conformers. The difference of $\Delta G^{\ddagger}$ calculated by applying the thermodynamic corrections of the minimum-energy conformer to all conformers of the state and using conformer-specific thermodynamic corrections varied from -0.3 to 1.2 kcal/mol among 8 macrocycles for which conformer-specific frequencies were calculated. The Cartesian coordinates of all minimum-energy conformers of the macrocycles are available from the data depository at https://doi.org/10.7924/r4474k10q.

All force-coupled stationary geometries and the attendant parameters (Supplementary Table 6) were optimized as previously described.[4,5] Force dependent electronic energies and constrained distances were obtained by interpolation of the results of relaxed potential energy scans of the $_{Me}C{\cdots}C_{Me}$ distance of $SS(OMe)_2$ of each conformer. Thermodynamic corrections were derived from frequency calculations on a subset of converged scan points. The use of analytical frequencies calculated on converged force-coupled geometries in this study is theoretically sound because the calculation is performed on the molecule plus its infinitely-compliant constraint (rather than just the molecule), which is a stationary point with all internal forces at 0.[6]

Supplementary Table 3. Notations used in presentations of computations and modeling. Calculations of these model parameters are described in the mechanochemical model of allosteric isomerization section below.

	Physical meaning	source
f_E, f_{TS}	Force exerted on the $_{MeO}C{\cdots}C_{OMe}$ coordinate of $SS(OMe)_2$ in a conformer of E or TS	f_E is the control parameter in our model; f_{TS} is calculated for each f_E and the compliance of the coupled potential as described below.
$\langle\rangle$	Ensemble averaging	$\langle X \rangle = \frac{\sum_{i=1}^{n} X_i e^{-\frac{E_i}{RT}}}{1+\sum_{i=1}^{n} e^{-\frac{E_i}{RT}}}$, where X_i is the quantity of interest in ith isomer, E_i is the energy of this isomer (relative to an arbitrary reference) and RT is thermal energy.
ΔE_{strain}	The change in the strain energy of the allosteric reactant due to effector binding	$\Delta E_{strain} = \Delta E_{mol} + \Delta E_{spr}$ where ΔE_{mol} is the change in ensemble-average molecular strain energy associated with a change in the ensemble-average force acting on the conformational ensemble of the E isomer from $\langle f_E \rangle$ to

		$\langle f_E' \rangle$. The force changes solely by the contraction of the equilibrium distance of the coupled constraining potential, which mimics effector binding. $\Delta E_{spr} = \lambda(\langle f_E \rangle^2 - \langle f_E' \rangle^2)$ is the associated increase in the potential energy of the coupled potential (spring; λ is the compliance). ΔE_{mol} and $\langle f_E' \rangle$ (for a given value of $\langle f_E \rangle$ and λ) are derived from DFT calculations (Supplementary Table 9).
$\Delta\Delta G^{o}_{bind}$	Standard reaction energy for transfer of $PtCl_2$ between BiPHEP and a macrocycle (rxn 1, main text)	DFT calculations
$\Delta\Delta G^{\ddagger}_{isom}$	Standard activation free energy of isomerization of E-SS in effector-bound allosteric reactant relative to free E-SS.	$\Delta\Delta G^{\ddagger}_{isom} = \Delta G^{\ddagger}_{allost} - \Delta G^{\ddagger}_{free}$; $\Delta G^{\ddagger}_{allost} = G_{TS}(\langle f_{TS} \rangle) - G_E(\langle f_E \rangle)$; $G_{TS}(\langle f_{TS} \rangle)$ and $G_E(\langle f_E \rangle)$ are derived from DFT calculations, and $\langle f_{TS} \rangle$ is calculated from $\langle f_E \rangle$ and λ; both procedures are described below.

In our benchmarking of different functional (u)BMK/6-31+G(d) reproduced experimental $\Delta G^{\ddagger}$ of three stiff-stilbene derivatives (Supplementary Table 4) most accurately. The sizes of the macrocycles in this work make their optimization at (u)BMK/6-31+G(d) untenable. The compromise reproduces $\Delta G^{\ddagger}$ for the macrocycles reported here (Table 1, main text), and elsewhere to within experimental accuracy and therefore accurately describes the electronic structure of these molecules.

Supplementary Table 4. Experimental and computed standard free-energies of activation of isomerization of Z-SS$(OMe)_2$ to E-SS$(OMe)_2$ or of 2 strained E macrocycles to their Z analogs for several functionals at 6-31+G* unless specified otherwise in vacuum. The UHF designation omitted for clarity.

reactant	exp	B3LYP	B3LYP[a]	B3LYP[b]	BMK	M06-2x	Cam-B3LYP	wB97x-D	MPW1K	APFD
Z-SS$(OMe)_2$	40±3[3]	35.2	36.2	40.0	38.9	42.2	35.3	36.5	31.8	33.6
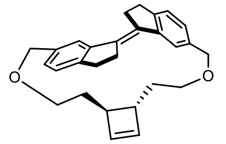	16.5±0.9[4]	13.6			17.2				12.1	
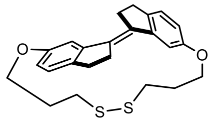	22±2[5]	19.1			21.7				15.0	

(a) def2SVP; (b) (u)BMK/def2SVP//(u)B3LYP/def2SVP

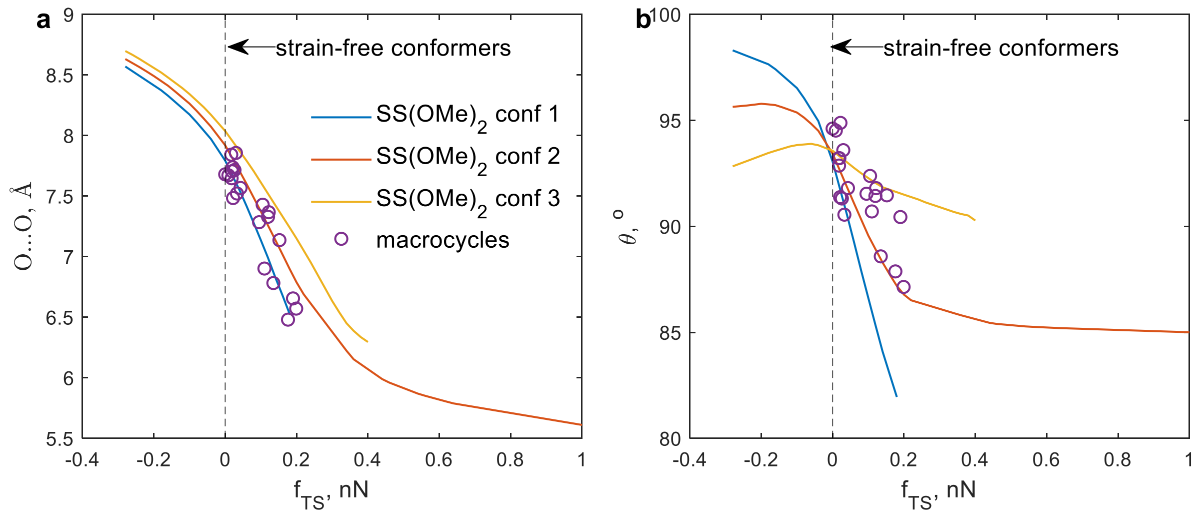

Supplementary Figure 28. The O···O distance and the torsion of the isomerizing C=C bond, θ, in thermally-accessible TS conformers of the macrocycles (dots) and in $SS(OMe)_2$ (lines). Compressive force is positive. The legend in (a) applies to (b) as well.

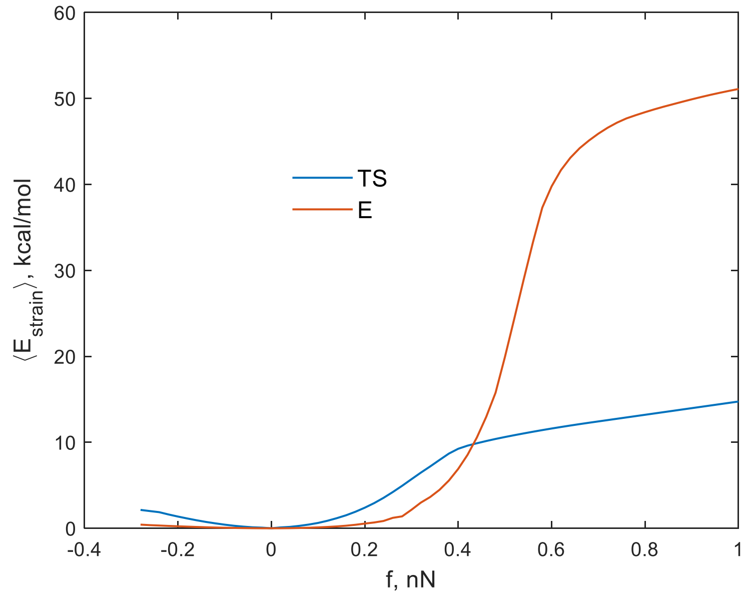

Supplementary Figure 29. Ensemble-average molecular strain energies of the conformational ensembles of $SS(OMe)_2$ coupled to an infinitely compliant potential (compressive for f>0 and tensile for f<0). Note the much smaller increase in the strain energy of the TS with compressive force, i.e., the TS is less destabilized by the compressive force than the E isomer.

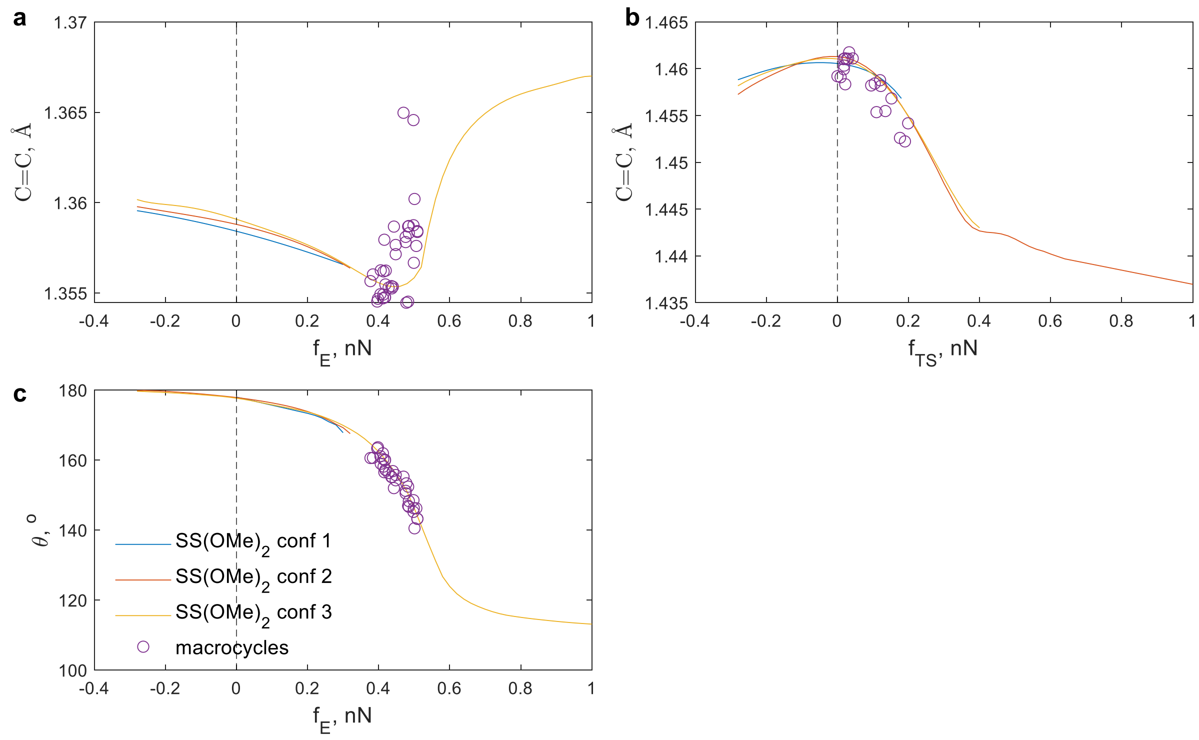

Supplementary Figure 30. The length of the isomerizable C=C bond in thermally accessible conformers of E-SS(OMe)$_2$ (lines) and macrocycles (dots) (a) and the equivalent data in the transition-state conformers (b). The torsion of the isomerizable C=C bond, θ, in thermally-accessible conformers of force-coupled E-SS(OMe)$_2$ (lines) and E macrocycles (dots). Compressive force is positive. The legend in (c) applies to (a-b) as well. The coupled potential is infinitely soft.

Supplementary Table 5. Standard reaction energies for rxn 1 (main text), $\Delta\Delta G^o_{bind}$, in kcal/mol

	(S,R)-E(2,2)	(S,S)-E(2,2)	(S,R)-E(2,3)	(S,S)-E(2,3)	(S,R)-E(3,3)	(S,S)-E(3,3)
$\Delta\Delta G^o_{bind}$	5.1	4.0	3.2	3.6	2.3	2.4

Supplementary Table 6. Ensemble-average force-dependent structural parameters and molecular strain energy of E-SS(OMe)$_2$ and the corresponding TS as a function of force generated by a hypothetical infinitely compliant harmonic constraint. Compressive force is positive. At f_E>340 pN and f_{TS}>400 pN the corresponding ensembles are comprised of a single conformer. See Fig. 2 of the main text for pictorial definitions of $_{MeO}C\ldots C_{OMe}$, $_{Me}O\ldots O_{Me}$ and θ coordinates. These data are derived directly from DFT scans.

	E isomer				TS			
Force, pN	E_{strain}, kcal/mol	$_{MeO}C\ldots C_{OMe}$, Å	$_{Me}O\ldots O_{Me}$, Å	θ °	E_{strain}, kcal/mol	$_{MeO}C\ldots C_{OMe}$, Å	$_{Me}O\ldots O_{Me}$, Å	θ °
-250	0.34	11.47288	10.58162	179.7	1.94	9.54792	8.607048	94.6
-200	0.23	11.42113	10.56402	179.5	1.36	9.325542	8.517479	94.7
-150	0.13	11.36929	10.54504	179.2	0.83	9.085572	8.416001	94.7
-100	0.06	11.31655	10.52414	178.8	0.42	8.842678	8.298154	94.6
-50	0.02	11.26082	10.50088	178.3	0.15	8.574499	8.149911	94.3
0	0.00	11.80568	10.41033	177.8	0.00	8.243746	7.974616	93.5
50	0.02	11.74292	10.372	176.9	0.22	7.830374	7.741514	92.2
100	0.10	11.68191	10.32995	176.0	0.62	7.304051	7.508895	91.1
150	0.25	11.61816	10.28505	174.9	1.34	6.721243	7.293167	90.7
200	0.54	11.56325	10.23201	173.6	2.39	6.155162	7.109051	91.0
250	1.03	11.45899	10.15158	171.8	3.87	5.662408	6.881547	91.0
300	2.15	11.01553	10.04445	169.0	5.71	5.168116	6.625626	90.8
350	4.05	9.510092	9.963857	166.9	7.57	4.750439	6.413404	90.5
400	6.90	8.991265	9.686543	162.4	9.25	4.407472	6.287787	90.1
450	11.76	8.210683	9.24293	155.8	13.32	3.708055	5.974509	85.4
500	19.92	7.043256	8.539028	146.3	13.91	3.627266	5.914552	85.3
550	31.08	5.57049	7.570356	133.7	14.43	3.554151	5.860298	85.3
600	39.77	4.486036	6.787548	123.9	14.90	3.499249	5.819478	85.2
650	43.65	4.043855	6.434644	119.7	15.33	3.450389	5.783178	85.2
700	45.90	3.804498	6.240751	117.4	15.73	3.417056	5.758532	85.2
750	47.43	3.650488	6.115899	115.9	16.11	3.383722	5.733886	85.1
800	48.40	3.561661	6.043996	115.1	16.50	3.350389	5.70924	85.1
850	49.18	3.495523	5.990499	114.5	16.89	3.317056	5.684594	85.1
900	49.90	3.4383	5.944239	113.9	17.27	3.283722	5.659949	85.1
950	50.53	3.389948	5.905269	113.5	17.66	3.250389	5.635303	85.0
1000	51.10	3.350677	5.873759	113.1	18.04	3.217056	5.610657	85.0

Supplementary Table 7. Activation free energy of E-SS(OMe)$_2$ isomerization ($\Delta G^{\ddagger}$ in kcal/mol), coupled to harmonic compressive potentials of different compliances (listed in the 2nd row in Å/nN) as a function of the ensemble-average force exerted on E-SS(OMe)$_2$, $\langle f_E \rangle$, and the ensemble-average force of the corresponding TS, $\langle f_{TS} \rangle$ (both in pN). Maximum $\langle f_E \rangle$ that a potential can apply increases with decreasing compliance of the potential: consequently, $\langle f_{TS} \rangle$ and $\Delta G^{\ddagger}$ is meaningless for soft potentials above threshold $\langle f_E \rangle$ and the corresponding cells are empty.

	$\langle f_{TS} \rangle$										$\Delta G^{\ddagger}$									
$\langle f_E \rangle$	100	20	10	7	5	3	2	1	0.3	0.1	100	20	10	7	5	3	2	1	0.3	0.1
0	-31	-65	-80	-91	-99	-108	-101	-85	-94	-95	40.8	41.8	41.9	41.9	41.8	41.9	41.9	41.4	41.4	42.2
50	18	-43	-53	-54	-55	-58	-63	-61	-72	-75	38.1	39.9	40.5	40.7	40.9	40.9	41.1	41.1	41.1	41.2
100	66	-19	-47	-48	-45	-41	-38	-41	-52	-55	35.5	37.8	39.2	39.7	40.0	40.2	40.4	40.7	40.7	40.7
150		15	-39	-49	-49	-44	-36	-31	-35	-39		35.7	37.6	38.5	39.0	39.6	39.9	40.2	40.4	40.4
200		54	-20	-46	-50	-50	-42	-29	-25	-28		33.5	35.8	37.2	37.9	38.7	39.3	39.8	40.0	40.1
250		95	11	-32	-47	-51	-50	-40	-27	-24		31.0	33.6	35.3	36.2	37.3	38.2	38.9	39.3	39.4
300		139	51	-4	-34	-50	-52	-54	-43	-36		28.1	31.0	32.8	34.0	35.4	36.6	37.6	38.2	38.4
350		186	97	40	1	-36	-50	-61	-58	-56		25.0	28.0	29.9	31.1	32.7	34.2	35.4	36.2	36.5
400		237	153	97	58	9	-30	-56	-60	-64		21.5	24.4	26.2	27.3	28.9	30.5	31.9	32.9	33.2
450			221	171	137	91	43	1	-20	-31			20.0	21.5	22.5	23.8	25.2	26.5	27.5	27.9
500			305	259	232	194	156	108	80	65			14.9	16.0	16.7	17.6	18.5	19.3	20.2	20.5
550				364	341	301	263	230	199	186				9.9	11.0	11.6	12.0	12.4	13.0	13.2
600				487	453	414	373	326	298	279				7.0	7.5	7.8	8.0	8.4	8.8	8.9
650					551	519	472	416	371	355					6.2	6.3	6.6	6.9	7.2	7.4
700					634	607	568	512	453	430					5.6	5.7	5.8	6.0	6.3	6.4
750						684	652	601	535	502						5.4	5.5	5.5	5.7	5.8
800						747	721	674	604	566						5.3	5.3	5.3	5.4	5.5
850						805	783	743	673	628						5.1	5.1	5.2	5.2	5.2
900						861	842	807	745	698						5.0	5.0	5.0	5.1	5.1
950						915	898	866	810	767						4.8	4.8	4.9	5.0	5.0
1000						967	950	920	866	826						4.6	4.7	4.7	4.8	4.9

Supplementary Table 8. Components of $\Delta G^{\ddagger}(\langle f_E \rangle)$: the difference in the ensemble-average molecular strain energy of $SS(OMe)_2$ in the transition and reactant states, ΔE_{mol}, and the corresponding difference in the potential energy of the coupled constraining potential, ΔE_{spring} both in kcal/mol. Small differences between $\Delta G^{\ddagger}$ and $\Delta G^{\ddagger}_o + \Delta E_{mol} + \Delta E_{spring}$ at any value of $\langle f_E \rangle$ are due to rounding of the values in the table Maximum $\langle f_E \rangle$ that a potential can apply increases with decreasing compliance of the potential: consequently, $\langle f_{TS} \rangle$ and $\Delta G^{\ddagger}$ is meaningless for soft potentials above threshold $\langle f_E \rangle$ and the corresponding cells are empty. $\Delta E_X < 0$ corresponds to stabilization of the TS relative to the reactant.

	ΔE_{mol}										ΔE_{spring}									
$\langle f_E \rangle$	100	20	10	7	5	3	2	1	0.3	0.1	100	20	10	7	5	3	2	1	0.3	0.1
0	0.1	1.3	1.6	1.6	1.6	1.5	1.4	1.5	1.6	2.2	0.7	0.5	0.3	0.3	0.2	0.5	0.5	-0.1	-0.1	0.0
50	-0.3	-0.1	0.5	0.7	0.8	0.9	1.0	1.1	1.1	1.2	-1.6	0.0	0.1	0.1	0.1	0.1	0.1	0.0	0.0	0.0
100	-0.5	-0.9	-0.4	0.0	0.1	0.4	0.5	0.7	0.7	0.7	-4.0	-1.3	-0.4	-0.2	-0.2	-0.1	-0.1	0.0	0.0	0.0
150		-1.2	-1.0	-0.6	-0.4	-0.1	0.2	0.3	0.4	0.4		-3.1	-1.4	-0.8	-0.6	-0.4	-0.2	-0.1	0.0	0.0
200		-1.3	-1.4	-1.1	-0.9	-0.5	-0.2	0.0	0.1	0.1		-5.2	-2.8	-1.7	-1.2	-0.8	-0.5	-0.2	-0.1	0.0
250		-1.5	-2.0	-1.9	-1.8	-1.4	-1.0	-0.7	-0.5	-0.5		-7.5	-4.4	-2.8	-2.0	-1.3	-0.8	-0.4	-0.1	0.0
300		-1.9	-2.8	-3.0	-2.9	-2.6	-2.2	-1.9	-1.6	-1.6		-10.0	-6.2	-4.2	-3.1	-2.0	-1.2	-0.6	-0.2	-0.1
350		-2.6	-4.0	-4.4	-4.6	-4.5	-4.1	-3.8	-3.5	-3.4		-12.4	-8.0	-5.7	-4.3	-2.8	-1.6	-0.8	-0.3	-0.1
400		-3.7	-5.9	-6.7	-7.2	-7.4	-7.3	-7.0	-6.8	-6.7		-14.8	-9.7	-7.1	-5.5	-3.8	-2.2	-1.1	-0.4	-0.1
450			-9.2	-10.4	-11.1	-11.6	-12.0	-12.1	-12.0	-12.0			-10.8	-8.1	-6.5	-4.5	-2.8	-1.4	-0.5	-0.1
500			-14.1	-15.4	-16.4	-17.4	-18.3	-19.0	-19.2	-19.3			-11.0	-8.6	-6.9	-5.0	-3.2	-1.6	-0.6	-0.2
550				-22.2	-22.5	-23.5	-24.8	-25.9	-26.4	-26.7				-7.9	-6.5	-4.9	-3.2	-1.7	-0.6	-0.2
600				-27.2	-27.2	-27.8	-28.9	-29.9	-30.6	-30.9				-5.9	-5.4	-4.4	-3.1	-1.7	-0.6	-0.2
650					-29.7	-30.0	-30.6	-31.4	-32.1	-32.4					-4.1	-3.6	-2.8	-1.7	-0.7	-0.2
700					-31.3	-31.5	-31.8	-32.4	-33.1	-33.4					-3.1	-2.8	-2.4	-1.6	-0.7	-0.2
750						-32.3	-32.6	-33.0	-33.6	-34.0						-2.2	-1.9	-1.4	-0.7	-0.2
800						-32.8	-33.0	-33.4	-34.0	-34.3						-1.9	-1.7	-1.3	-0.7	-0.2
850						-33.2	-33.3	-33.6	-34.2	-34.5						-1.7	-1.5	-1.2	-0.6	-0.2
900						-33.4	-33.6	-33.8	-34.3	-34.7						-1.6	-1.4	-1.1	-0.6	-0.2
950						-33.7	-33.8	-34.0	-34.5	-34.8						-1.5	-1.4	-1.1	-0.6	-0.2
1000						-33.8	-33.9	-34.2	-34.6	-34.8						-1.6	-1.4	-1.1	-0.6	-0.2

Supplementary Table 9. The minimum required increase in the strain energy of the allosteric reactant upon effector binding, ΔE_{strain}, needed to achieve the desired lowering of the isomerization barrier, $\Delta\Delta G^{\ddagger}_{iso}$ (both in kcal/mol) and the minimum degree of pre-strain of the reactive moiety (stiff stilbene in our example) in the effector-free reactant required to achieve the given combination of ΔE_{strain} and $\Delta\Delta G_{iso}$, as quantified by its $\langle f_E \rangle$. Both parameters are quantified for constraining potentials of different compliance (shown in the 2nd row in Å/nN). $\langle f_E \rangle$=0 means that the substrate doesn't require prestraining to achieve maximum $\Delta\Delta G_{iso}$/ ΔE_{strain} ratio. The maximum achievable barrier lowering increases with decreasing potential compliance: empty cells correspond to barrier lowering that is unachievable with the specified compliance regardless of how much strain effector binding imposes on the substrate.

	ΔE_{strain}										$\langle f_E \rangle$, pN									
$\Delta\Delta G^{\ddagger}_{iso}$	100	20	10	7	5	3	2	1	0.3	0.1	100	20	10	7	5	3	2	1	0.3	0.1
-37						61.5	58.3	57.9	55.2	54.8						0	0	0	0	0
-36.5						59.1	56.0	55.9	53.4	52.9						0	0	0	0	0
-36						56.8	53.9	54.0	51.7	51.0						0	0	0	0	0
-35.5					59.8	54.5	51.9	52.2	50.0	49.2					0	0	0	0	0	0
-35					57.5	52.4	49.9	50.4	48.4	47.5					0	0	0	0	0	0
-34.5					55.4	50.3	48.0	48.7	46.9	45.8					0	0	0	0	0	0
-34				58.0	53.3	48.4	46.2	47.0	45.4	44.2				0	0	0	0	0	0	0
-33.5				55.7	51.3	46.5	44.5	45.4	43.9	42.7				0	0	0	0	0	0	0
-33				53.6	49.4	44.7	42.9	43.9	42.5	41.2				0	0	0	0	0	0	0
-32.5				51.5	47.5	43.0	41.3	42.4	41.1	39.8				0	0	0	0	0	0	0
-32				49.5	45.8	41.4	39.8	40.9	39.8	38.5				0	0	0	0	0	0	0
-31.5				47.6	44.1	39.8	38.4	39.6	38.5	37.2				0	0	0	0	0	0	0
-31				45.8	42.5	38.4	37.0	38.2	37.3	35.9				0	0	0	0	0	0	0
-30.5				44.1	40.9	36.9	35.7	36.9	36.1	34.7				0	0	0	0	0	0	0
-30				42.4	39.4	35.6	34.5	35.7	34.9	33.6				0	0	0	0	0	0	0
-29.5				40.8	38.0	34.3	33.3	34.5	33.8	32.5				0	0	0	0	0	0	0
-29			42.1	39.3	36.6	33.1	32.1	33.4	32.7	31.4			0	0	0	0	0	0	0	0
-28.5			40.8	37.8	35.3	31.9	31.0	32.2	31.7	30.4			0	0	0	0	0	0	0	0
-28			39.7	36.4	34.0	30.8	30.0	31.2	30.7	29.5			0	0	0	0	0	0	0	2
-27.5			38.5	35.1	32.8	29.7	29.0	30.1	29.7	28.5			0	0	0	0	0	0	0	5
-27			37.3	33.8	31.7	28.7	28.0	29.1	28.8	27.6			0	0	0	0	0	0	0	11
-26.5			36.2	32.6	30.6	27.7	27.1	28.2	27.8	26.8			0	0	0	0	0	0	0	17
-26			35.0	31.4	29.5	26.7	26.2	27.3	27.0	26.0			0	0	0	0	0	0	0	19
-25.5			33.9	30.3	28.5	25.8	25.3	26.4	26.1	25.2			0	0	0	0	0	0	0	19
-25			32.8	29.2	27.5	24.9	24.5	25.5	25.3	24.4			0	0	0	0	0	0	0	19
-24.5			31.8	28.2	26.5	24.1	23.7	24.7	24.5	23.7			0	0	0	0	0	0	0	20
-24			30.7	27.2	25.6	23.3	23.0	23.9	23.7	23.0			0	0	0	0	0	0	0	20

$\Delta\Delta G^{\ddagger}_{iso}$	ΔE_{strain}										$\langle f_E \rangle$, pN									
	100	20	10	7	5	3	2	1	0.3	0.1	100	20	10	7	5	3	2	1	0.3	0.1
-23.5			29.7	26.2	24.7	22.5	22.3	23.1	22.9	22.3			0	0	0	0	0	0	0	20
-23			28.6	25.3	23.8	21.8	21.6	22.3	22.2	21.6			0	0	0	0	0	0	0	20
-22.5			27.6	24.4	23.0	21.1	20.9	21.6	21.5	21.0			0	0	0	0	0	0	0	20
-22			26.6	23.5	22.2	20.4	20.2	20.9	20.8	20.4			0	0	0	0	0	0	0	20
-21.5		33.1	25.7	22.6	21.4	19.7	19.6	20.2	20.2	19.8		0	0	0	0	0	0	0	0	20
-21		31.8	24.7	21.8	20.7	19.1	19.0	19.6	19.5	19.2		0	0	0	0	0	0	0	0	20
-20.5		30.5	23.8	21.0	19.9	18.4	18.4	19.0	18.9	18.6		0	0	0	0	0	0	0	0	20
-20		29.3	22.9	20.3	19.2	17.8	17.8	18.3	18.3	18.1		0	0	0	0	0	0	0	0	20
-19.5		28.1	22.0	19.5	18.5	17.2	17.2	17.7	17.7	17.5		0	0	0	0	0	0	0	0	20
-19		26.8	21.1	18.8	17.9	16.6	16.7	17.2	17.1	17.0		0	0	0	0	0	0	0	0	20
-18.5		25.7	20.2	18.1	17.2	16.1	16.1	16.6	16.6	16.5		0	0	0	0	0	0	0	0	20
-18		24.5	19.3	17.4	16.6	15.5	15.6	16.1	16.0	16.0		0	0	0	0	0	0	0	0	20
-17.5		23.4	18.5	16.7	16.0	15.0	15.1	15.5	15.5	15.5		0	0	0	0	0	0	0	0	20
-17		22.3	17.7	16.1	15.3	14.4	14.6	15.0	15.0	15.0		0	0	0	0	0	0	0	0	20
-16.5		21.2	16.9	15.4	14.8	13.9	14.1	14.5	14.5	14.6		0	0	0	0	0	0	0	0	22
-16		20.1	16.1	14.8	14.2	13.4	13.6	14.0	14.0	14.1		0	0	0	0	0	0	0	2	27
-15.5		19.1	15.3	14.2	13.6	12.9	13.1	13.5	13.5	13.6		0	0	0	0	0	0	0	7	43
-15		18.1	14.6	13.6	13.0	12.4	12.6	13.0	13.0	13.2		0	0	0	0	0	0	0	13	85
-14.5		17.1	13.8	13.0	12.5	11.9	12.1	12.6	12.6	12.7		0	0	0	0	0	0	0	15	162
-14		16.2	13.1	12.4	11.9	11.4	11.7	12.1	12.1	12.3		0	0	0	0	0	0	0	18	244
-13.5		15.3	12.4	11.8	11.4	10.9	11.2	11.7	11.6	11.8		0	0	0	0	0	0	0	25	292
-13		14.4	11.7	11.2	10.9	10.4	10.7	11.2	11.2	11.4		0	0	0	0	0	0	0	47	311
-12.5		13.5	11.1	10.7	10.3	10.0	10.3	10.8	10.8	11.0		0	0	0	0	0	0	1	99	317
-12		12.6	10.4	10.1	9.8	9.5	9.8	10.3	10.3	10.5		0	0	0	0	0	0	6	180	320
-11.5		11.8	9.8	9.5	9.3	9.0	9.3	9.9	9.9	10.1		0	0	0	0	0	0	9	249	320
-11		11.0	9.2	9.0	8.8	8.5	8.9	9.5	9.5	9.6		0	0	0	0	0	0	17	283	320
-10.5		10.2	8.6	8.5	8.3	8.1	8.4	9.1	9.0	9.2		0	0	0	0	0	0	37	296	320
-10		9.5	8.0	7.9	7.8	7.6	8.0	8.6	8.6	8.8		0	0	0	0	0	0	79	300	320
-9.5		8.8	7.4	7.4	7.3	7.2	7.5	8.2	8.2	8.3		0	0	0	0	0	0	143	302	320
-9		8.1	6.9	6.9	6.9	6.7	7.1	7.8	7.8	7.9		0	0	0	0	0	0	207	304	320
-8.5		7.4	6.4	6.4	6.4	6.3	6.7	7.4	7.4	7.5		0	0	0	0	0	0	248	305	320
-8		6.8	5.9	5.9	5.9	5.8	6.2	7.0	7.0	7.0		0	0	0	0	0	0	267	306	320
-7.5		6.2	5.4	5.4	5.5	5.4	5.8	6.6	6.6	6.6		0	0	0	0	0	0	275	308	320
-7		5.6	4.9	5.0	5.0	5.0	5.3	6.1	6.1	6.2		0	0	0	0	0	0	278	310	320

$\Delta\Delta G^{\ddagger}_{iso}$	ΔE_{strain}										$\langle f_E \rangle$, pN									
	100	20	10	7	5	3	2	1	0.3	0.1	100	20	10	7	5	3	2	1	0.3	0.1
-6.5		5.0	4.4	4.5	4.6	4.5	4.9	5.7	5.7	5.7		0	0	0	0	0	0	279	312	320
-6		4.5	4.0	4.1	4.2	4.1	4.5	5.3	5.3	5.3		0	0	0	0	0	0	280	315	320
-5.5		3.9	3.6	3.6	3.8	3.7	4.1	4.9	4.9	4.8		0	0	0	0	0	0	280	317	321
-5	6.5	3.5	3.2	3.2	3.3	3.3	3.7	4.4	4.5	4.4	0	0	0	0	0	0	0	280	320	321
-4.5	5.2	3.0	2.8	2.8	2.9	2.9	3.2	4.0	4.0	4.0	0	0	0	0	0	0	0	280	323	322
-4	4.1	2.6	2.4	2.4	2.6	2.5	2.8	3.6	3.6	3.5	0	0	0	0	0	0	0	280	326	325
-3.5	3.1	2.2	2.0	2.0	2.2	2.2	2.5	3.2	3.2	3.1	0	0	0	0	0	0	0	280	329	331
-3	2.3	1.8	1.7	1.7	1.8	1.8	2.1	2.7	2.7	2.6	0	0	0	0	0	0	0	280	332	337
-2.5	1.6	1.4	1.4	1.3	1.5	1.5	1.7	2.3	2.3	2.2	0	0	0	0	0	0	0	280	334	339
-2	1.0	1.1	1.1	1.0	1.1	1.1	1.3	1.8	1.8	1.7	1	4	3	2	3	4	4	280	336	340
-1.5	0.6	0.8	0.8	0.7	0.8	0.8	1.0	1.4	1.4	1.3	5	14	13	11	16	21	23	281	338	341
-1	0.3	0.5	0.5	0.4	0.5	0.5	0.6	0.9	0.9	0.9	21	37	41	42	64	84	98	290	340	341
-0.5	0.1	0.2	0.2	0.2	0.3	0.3	0.3	0.5	0.5	0.4	49	64	73	85	132	150	202	298	341	341

Synthesis of bisphosphine ligands

(*S,S*)-E(3,3) and (*R,S*)-E(3,3). A solution of Z(3,3)[7] (198 mg, 0.220 mmol) in CH_2Cl_2 (200 mL) was sparged with argon for 30 min and then irradiated with a 3W 365 nm single diode high-powered LED (~17.5 mW cm^{-2}) in contact with the bottom of the flask for 40 min to form a photo-stationary 68:23:9 mixture of Z(3,3)**,** (*S,S*)-E(3,3), and (*R,S*)-E(3,3) . The solvent was evaporated under vacuum and the resulting white solid was chromatographed (SiO_2, gradient hexanes - 80:20 hexanes/EtOAc) to give Z(3,3) (143 mg, 72%), (*S,S*)-E(3,3) (35 mg, 18%), and (*R,S*)-E(3,3) (11 mg, 6%) with 95% overall recovery. Ligands (*R,S*)-E(2,3) and E(2,2) were synthesized from Z(2,3)[1] and Z(2,2),[1] respectively, employing analogous procedures.

For (*S,S*)-E(3,3): ^{1}H NMR (400 MHz, $CDCl_3$): δ 7.25–7.08 (m, 14H), 7.08–6.96 (m, 6H), 6.82 (d, *J* = 2.3 Hz, 2H), 6.74 (t, *J* = 6.6 Hz, 4H), 6.69 (d, *J* = 8.1 Hz, 4H), 6.65 (dd, *J* = 8.1, 2.1 Hz, 2H), 4.02–3.77 (m, 4H), 3.50–3.38 (m, 2H), 3.31–3.20 (m, 2H), 2.96 (dd, *J* = 13.2, 7.3 Hz, 2H), 2.91–2.72 (m, 6H), 1.34– 1.22 (m, 2H), 0.88–0.71 (m, 2H). ^{13}C{^{1}H} NMR (126 MHz, $CDCl_3$): δ 156.99, 155.46, 144.99, 141.45, 139.05, 138.56, 134.98, 133.96, 133.79, 133.13, 132.97, 128.41, 128.15, 127.65, 127.08, 125.99, 118.31, 113.12, 111.02, 66.75, 64.97, 35.64, 31.78, 26.60. ^{31}P NMR (162 MHz, $CDCl_3$): δ –14.97. HRMS-ESI (m/z) calcd (found) for $C_{60}H_{52}O_4P_2$) [MH]$^+$: 899.3414 (899.3176).

For (*R,S*)-E(3,3): ^{1}H NMR (500 MHz, $CDCl_3$): δ 7.37–7.23 (m, 12H), 7.17–7.07 (m, 8H), 7.02 (d, *J* = 1.4 Hz, 2H), 6.91-6.88 (m, 4H), 6.76 (dd, *J* = 8.1, 1.7 Hz, 2H), 6.70 (dd, *J* = 7.6, 2.8 Hz, 2H), 6.13 (d, *J* = 8.2 Hz, 2H), 4.11–4.01 (m, 2H), 3.81–3.70 (m, 2H), 3.21–3.02 (m, 3H), 3.01–2.86 (m, 3H), 1.41–1.19 (m, 4H). ^{13}C{^{1}H} NMR (126 MHz, CD_2Cl_2): δ 156.8 (d, *J* = 6.1 Hz), 156.7 (d, *J* = 6.1 Hz), 156.4, 144.8, 142.0, 139.2 (d, *J* = 9.2 Hz), 138.9 (d, *J* = 4.7 Hz), 137.9 (d, *J* = 9.3 Hz), 134.4 - 134.1 (m), 132.9 - 133.0 (m), 128.3 - 128.8 (m), 128.1, 119.0, 113.0, 111.1, 67.2, 65.8, 35.3, 31.6, 28.7. ^{31}P NMR (162 MHz, $CDCl_3$): δ –15.09. HRMS-ESI (m/z) calcd (found) for $C_{60}H_{52}O_4P_2$ [MH]$^+$: 899.3414 (899.3411).

(*R,S*)-E(2,3).[1] Macrocycle (*R,S*)-E(2,3) was isolated in 58% yield as a white solid from a photo-stationary 42:58 mixture of Z(2,3) and (*R,S*)-E(2,3). ^{1}H NMR (500 MHz, CD_2Cl_2): δ 7.47 – 7.34 (m, 6H), 7.32 – 7.25 (m, 5H), 7.22 (td, *J* = 8.0, 6.6 Hz, 2H), 7.15 – 7.05 (m, 6H), 6.99 (td, *J* = 7.6, 1.7 Hz, 2H), 6.88 – 6.76 (m, 7H), 6.64 (dd, *J* = 8.2, 2.3 Hz, 1H), 6.53 (tt, *J* = 6.7, 1.4 Hz, 2H), 6.05 (d, *J* = 8.3 Hz, 1H), 3.98 – 3.89 (m, 3H), 3.77 – 3.69 (m, 1H), 3.58 – 3.48 (m, 2H), 3.06 – 2.65 (m, 8H), 2.39 – 2.28 (m, 1H), 1.69 (ddd, *J* = 13.5, 9.2, 4.5 Hz, 1H), 1.11 (tdd, *J* = 14.0, 9.2, 4.4 Hz, 1H), 0.44 – 0.33 (m, 1H). ^{13}C{^{1}H} NMR: (126 MHz, CD_2Cl_2): δ 157.59, 157.51, 157.36, 156.97, 156.88, 155.30, 146.15, 145.05, 144.05, 140.72, 140.42, 140.30, 139.53, 139.48, 139.30, 139.21, 138.68, 138.56, 138.50, 138.30, 138.19, 135.66, 135.08, 134.10, 134.07, 133.94, 133.91, 133.33, 133.18, 132.87, 132.72, 128.99, 128.94, 128.92, 128.78, 128.73, 128.65, 128.62, 128.57, 128.53, 128.48, 128.34, 128.31, 128.27, 128.19, 127.74, 127.59, 126.77, 125.70, 120.74, 120.14, 118.42, 113.04, 111.90, 108.73, 70.78, 68.14, 67.97, 65.44, 35.01, 32.14, 31.65, 28.73. ^{31}P NMR (202 MHz, CD_2Cl_2): δ –14.61, –14.99. HRMS-ESI (m/z) calcd (found) for $C_{59}H_{50}O_4P_2$ [MH]$^+$: 885.3179 (885.3267).

(*R,S*)-E(2,2).[1] Macrocycle E(2,2) was isolated in 3.3% yield as a white solid from a photo-stationary 96:4 mixture of Z(2,2) and (*R,S*)-E(2,2). ^{1}H NMR (400 MHz, $CDCl_3$): δ 7.33 – 7.23 (m, 6H), 7.24 (d, *J* = 4.9 Hz, 1H), 7.2 (dd, *J* = 5.0, 2.8 Hz, 3H), 7.14 (d, *J* = 8.0 Hz, 2H), 7.06 (t, *J* = 7.3 Hz, 2H), 7.04 –6.93 (m, 8H), 6.91 (d, *J* = 8.2 Hz, 2H), 6.85 (dd, *J* = 7.6, 3.2 Hz, 2H), 6.67 – 6.54 (m, 6H), 4.44 (dd, *J* = 7.1, 14.1, 2 H), 4.28 (dd, *J* = 5.0, 13.7 Hz, 2 H) 3.77 (dd, *J* = 5.1, 14.3 Hz, 2 H), 3.73 (dd, *J* = 6.6, 13.8 Hz, 2 H), 2.97 (td, *J* = 7.0, 13.7 Hz, 2H), 2.87 (m, 4H), 2.74 (br d, *J* = 13.7 Hz, 2H). ^{13}C{^{1}H} NMR: (126 MHz, CD_2Cl_2): 159.1, 159.0, 157.2, 145.4, 141.8, 139.5, 139.0 (d, *J* = 15.4 Hz), 138.8 (d, *J* = 14.8 Hz), 138.5 (d, *J* = 10.3 Hz), 134.7, 133.2 (d, *J* = 19.8 Hz), 132.7 (d, *J* = 17.2 Hz), 129.8, 128.4 (d, *J* = 5.0 Hz), 128.2 (d, *J* = 6.4 Hz), 128.1 (d, *J* = 4.1 Hz), 127.8, 124.8, 118.8, 116.5, 113.8, 75.9, 71.4, 36.0, 29.8. ^{31}P NMR (162 MHz, $CDCl_3$): δ –10.71. HRMS-ESI (m/z) calcd (found) for $C_{58}H_{48}O_4P_2$ [MH]$^+$: 871.3101 (871.3098).

Supplementary References

1 Yu, Y. *et al.* Force-modulated reductive elimination from platinum(ii) diaryl complexes. *Chemical Science* **12**, 11130-11137 (2021). https://doi.org:10.1039/d1sc03182a

2 Chan, A. P. Y. *et al.* Selective ortho-C–H Activation in Arenes without Functional Groups. *Journal of the American Chemical Society* **144**, 11564-11568 (2022). https://doi.org:10.1021/jacs.2c04621

3 Cramer, C. J. *Essentials of Computational Chemistry*. 2nd ed. edn, (Wiley, 2004).

4 Akbulatov, S. *et al.* Experimentally realized mechanochemistry distinct from force-accelerated scission of loaded bonds. *Science* **357**, 299-303 (2017).

5 Boulatov, R. & O'Neill, R. T. Experimental quantitation of molecular conditions responsible for flow-induced mechanochemistry. *Nature Chemistry* **in press** (2023).

6 Kucharski, T. J. & Boulatov, R. The physical chemistry of mechanoresponsive polymers. *J. Mater. Chem.* **21**, 8237-8255 (2011). https://doi.org:10.1039/c0jm04079g

7 Kean, Z. S. *et al.* Photomechanical Actuation of Ligand Geometry in Enantioselective Catalysis. *Angew. Chem. Intl. Ed.* **53**, 14508-14511 (2014).
